# Three in One—Multiple Faunal Elements within an Endangered European Butterfly Species

**DOI:** 10.1371/journal.pone.0142282

**Published:** 2015-11-13

**Authors:** Marius Junker, Marie Zimmermann, Ana A. Ramos, Patrick Gros, Martin Konvička, Gabriel Nève, László Rákosy, Toomas Tammaru, Rita Castilho, Thomas Schmitt

**Affiliations:** 1 Department of Biogeography, Trier University, Trier, Germany; 2 Université de Tours, CNRS, UMR 6035 –IRBI, Avenue Monge, Parc Grandmont, Tours, France; 3 Centre of Marine Sciences, CCMAR/CIMAR Associate Laboratory, University of Algarve, Gambelas, Faro, Portugal; 4 Haus der Natur, Museum für Natur und Technik, Museumsplatz 5, Salzburg, Austria; 5 School of Biological Sciences, University South Bohemia, Branisovska 31, Ceske Budejovice, Czech Republic; 6 Institut Méditerranéen de Biodiversité et d’Ecologie marine et continentale, Aix Marseille Université, CNRS, IRD, Avignon Université, Case 36, 3 place Victor Hugo, Marseille Cedex 3, France; 7 Faculty of Biology, University Babes-Bolyai, Str. Clinicilor 5–7, Cluj, Romania; 8 Institute of Ecology and Earth Sciences, University of Tartu, Vanemuise 46, Tartu, Estonia; 9 Senckenberg German Entomological Institute, Eberswalder Straße 90, Müncheberg, Germany; 10 Zoology, Institute of Biology, Faculty Natural Science I, Martin Luther University Halle-Wittenberg, Halle (Saale), Germany; Oxford Brookes University, UNITED KINGDOM

## Abstract

Ice ages within Europe forced many species to retreat to refugia, of which three major biogeographic basic types can be distinguished: "Mediterranean", "Continental" and "Alpine / Arctic" species. However, this classification often fails to explain the complex phylogeography of European species with a wide range of latitudinal and altitudinal distribution. Hence, we tested for the possibility that all three mentioned faunal elements are represented within one species. Our data was obtained by scoring 1,307 *Euphydryas aurinia* individuals (46 European locations) for 17 allozyme loci, and sequencing a subset of 492 individuals (21 sites) for a 626 base pairs COI fragment. Genetic diversity indices, *F* statistics, hierarchical analyses of molecular variance, individual-based clustering, and networks were used to explore the phylogeographic patterns. The COI fragment represented 18 haplotypes showing a strong geographic structure. All but one allozyme loci analysed were polymorphic with a mean *F*_ST_ of 0.20, supporting a pronounced among population structure. Interpretation of both genetic marker systems, using several analytical tools, calls for the recognition of twelve genetic groups. These analyses consistently distinguished different groups in Iberia (2), Italy, Provence, Alps (3), Slovenia, Carpathian Basin, the lowlands of West and Central Europe as well as Estonia, often with considerable additional substructures. The genetic data strongly support the hypothesis that *E*. *aurinia* survived the last glaciation in Mediterranean, extra-Mediterranean and perialpine refugia. It is thus a rare example of a model organism that combines attributes of faunal elements from all three of these sources. The observed differences between allozymes and mtDNA most likely result from recent introgression of mtDNA into nuclear allozyme groups. Our results indicate discrepancies with the morphologically-based subspecies models, underlining the need to revise the current taxonomy.

## Introduction

Climatic oscillations with ice ages and interglacial periods have had strong impacts on the distribution of European animal and plant species (e.g. [1,2]). In this context, glacial refugia with suitable habitat conditions were pivotal for survival during glacial cycles [3]. Considering the geographic location of these refugia and the respective postglacial range changes, molecular analyses show that European species may be divided into three major biogeographic types: Mediterranean, continental and arctic/alpine [2]:

1. “Mediterranean” species usually had important glacial differentiation centres at least in one of the three major Mediterranean peninsulas, but also in the Maghreb and Asia Minor [4,5]. However, further substructures in these refugia have been postulated since the 1950s [6], and recent phylogeographic analyses support these old postulates well [2].
2. “Continental” species are supposed to have survived during ice ages in extra-Mediterranean refugia [7] with more buffered climatic conditions (e.g. the Carpathian Basin) [8].
3. “Arctic” and/or “Alpine” species often survived ice ages in perialpine refugia and retreated to alpine and/or arctic areas when temperatures increased after their inter-/postglacial deglaciation [9,10].

This classification is simplistic and often fails to explain the rather complex phylogeography of European species with a currently wide range of latitudinal and altitudinal distribution. Thus, a number of continental elements also had important glacial refugia in the Balkan Peninsula (e.g. several butterfly species [11,12]; the adder *Vipera berus* [13]; the slug *Arion fuscus* [14]). Mediterranean and extra-Mediterranean refugia existed in close geographic proximity in the Balkan Peninsula so that these continental species may have occurred in extra-Mediterranean refugia of this region [8]. Several alpine species apparently have co-occurred with continental elements in several ice age retreats in the vicinity of high mountain systems [8,10]. Furthermore, Mediterranean elements turned out to have had more diverse ice age refugia than previously thought, and many examples demonstrate extra-Mediterranean refugia in addition to the classical Mediterranean retreats [8,15]. This underlines the great biogeographic importance of the cryptic extra-Mediterranean refugia that although geographically smaller in size than the Mediterranean refugia are a reservoir of genetic variation and often play a leading edge colonization function [16,17]. Various species combine two of the biogeographic patterns mentioned above [8], but the most complex possible combination of Mediterranean, extra-Mediterranean and perialpine refugia has not been observed so far.

Therefore, we selected a particularly widespread and ecologically diverse species as study object, the fritillary *Euphydryas aurinia* (Rottemburg, 1775) (Nymphalidae: Melitaeinae). This species is a univoltine butterfly found from Mediterranean shrub-lands at sea level to alpine meadows, and from the European Atlantic coast throughout temperate Asia [18]. It colonizes a great variety of different habitats, e.g. open *Quercus* woodlands in the Iberian Peninsula (*E*. *aurinia beckeri* (Herrich-Schäffer, 1851)) [19,20], dry or damp calcareous or acidophilic grasslands and mires in Central Europe and the UK (*E*. *aurinia aurinia* Rottemburg, 1775) [21–24] as well as nutrient-poor alpine meadows in the Pyrenees (*E*. *aurinia debilis* Oberthür, 1909) and the Alps (*E*. *aurinia glaciegenita* Verity, 1928) [18,25,26]. Furthermore, different larval food plants are used regionally, including the genera *Lonicera*, *Succisa*, *Scabiosa* and *Gentiana* [27,28]. Due to this wide variation of ecological constraints and morphological adaptations, many subspecies have been described in the Palaearctic region[29]; recent reviews mention 12 subspecies for the western Palaearctic [30], with six subspecies for France alone [28]

*Euphydryas aurinia* is highly threatened in Central Europe due to dramatic damage of its habitats during the last few decades [31–33] and hence was listed in Annex II of the European Habitats Directive. Since then, important efforts have been made to collect knowledge that will aid conservation, especially of the populations in western and northern Europe [23,34–36]. By contrast, populations in Iberia and southern France are considered mostly stable [28,37].

Due to these strong ecological adaptations, it seemed possible that *E*. *aurinia* may represent, within a single species, all three major biogeographic groups known for Europe (cf. [2]). By applying mitochondrial and nuclear markers to populations sampled over most of the European range of *E*. *aurinia*, we analyse the differentiation patterns within this species; hereby we aim at identifying the location of glacial refugia and the postglacial range changes.

## Material and Methods

### Sampling

**Fig 1:**
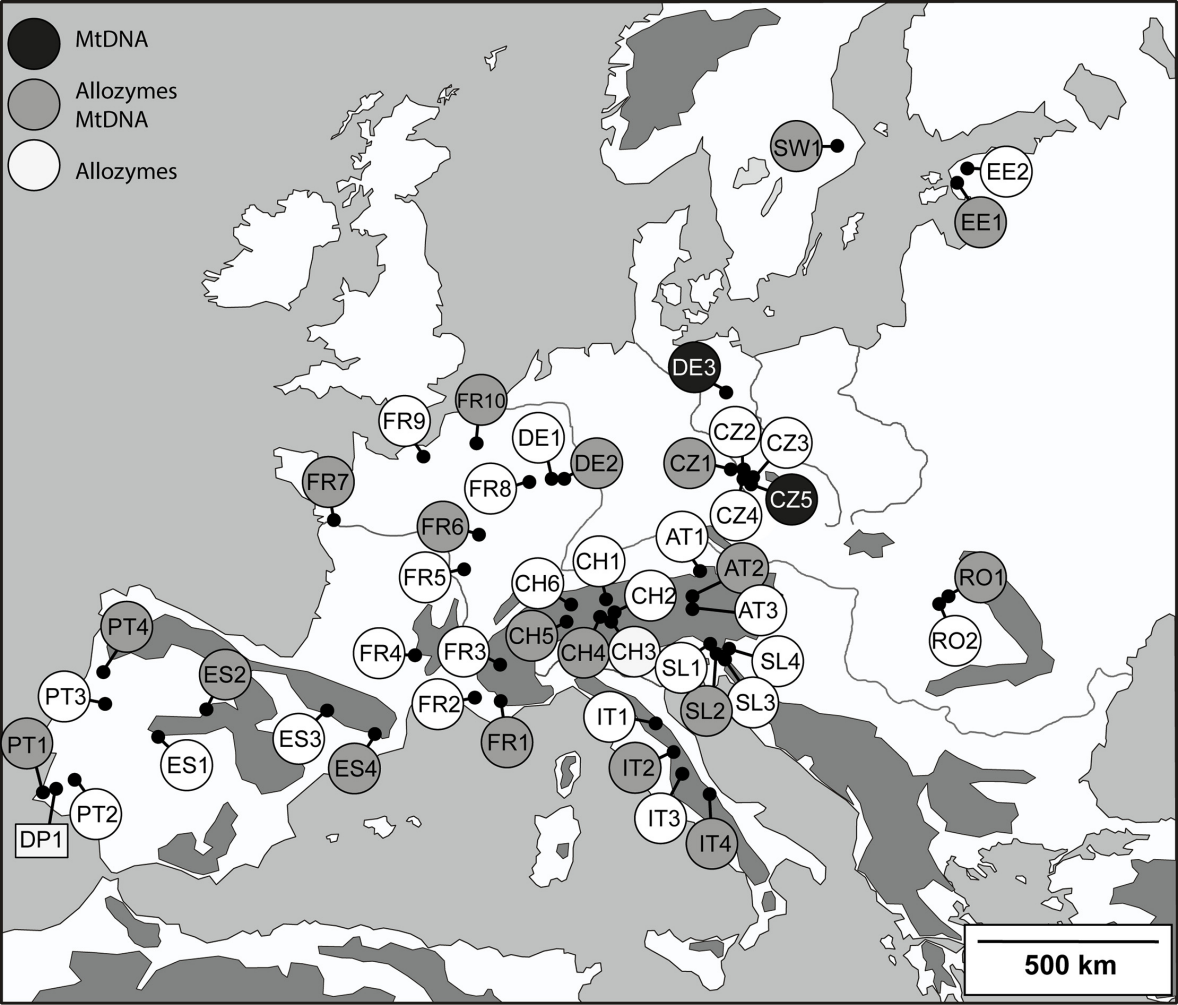
Localities of the 48 sampling sites of *Euphydryas aurinia* and one sampling site of *Euphydryas desfontainii* (DP1, outgroup) for genetic analyses. For abbreviations see Table 1.

We sampled 1,307 *E*. *aurinia* individuals (46 populations) distributed over major parts of Europe for allozyme electrophoresis (Table 1; Fig 1). One sample of *Euphydryas desfontainii* (30 individuals) from southern Portugal was included as outgroup (sister species of *E*. *aurinia* [38]). The sample sizes ranged from 5 to 44 individuals (mean 28.4 ± 10.3 SD); only eight samples contained less than 20 individuals. After netting in the field, butterflies were immediately frozen in liquid nitrogen and stored in this medium until analysis. 492 of these individuals and from two additional sites were used for mtDNA sequencing.

**Table 1:** Sampling locations of *Euphydryas aurinia* and *E*. *desfontainii* for mtDNA and allozyme analyses; presented in alphabetic order of countries. The mentioned subspecies are those recognised by Tshikolovets [30].

Code	CTY	Location	N	Capture date	Elevation (m a.s.l.)	Latitude	Longitude	Subspecies
AT1	AT	Wartberg	0/23	02.VI.2008	450	47° 45’ N	12° 56’ E	*aurinia*
AT2	AT	Oberes Nassfeld	25/43	24.VI.2007	2300	47° 07’ N	12° 49’ E	*glaciegenita*
AT3	AT	Schöneck	0/25	17.VII.2007	2100	47° 03’ N	12° 47’ E	*glaciegenita*
CH1	CH	Partnun	0/13	18.VII.2006	1800	46° 59’ N	09° 51’ E	*glaciegenita*
CH2	CH	Ofenpass	0/27	09.VII.2008	2400	46° 38’ N	10° 19’ E	*glaciegenita*
CH3	CH	Berninapass	0/36	10.VII.2008	2300	46° 24’ N	10° 01’ E	*glaciegenita*
CH4	CH	Julierpass	25/35	10.VII.2008	2300	46° 28’ N	09° 43’ E	*glaciegenita*
CH5	CH	Simplonpass	23/23	11.VII.2008	2000	46° 15’ N	08° 02’ E	*glaciegenita*
CH6	CH	Furkapass	0/26	11.VII.2008	2400	46° 34’ N	08° 24’ E	*glaciegenita*
CZ1	CZ	Trojmezi	25/25	VI.2007	600	50° 18' N	12° 06' E	*aurinia*
CZ2	CZ	Soos	0/24	VI.2007	450	50° 08' N	12° 25' E	*aurinia*
CZ3	CZ	Dominova skalka	0/23	VI.2007	750	50° 04' N	12° 46' E	*aurinia*
CZ4	CZ	Horní Kramolín	0/25	VI.2007	700	49° 58' N	12° 48' E	*aurinia*
CZ5	CZ	Krásno	30/0	VI.2007	800	50° 05' N	12° 44' E	*aurinia*
DE1	DE	Badstube	0/33	29.V.2008	250	49° 13’ N	07° 16’ E	*aurinia*
DE2	DE	Lohe	25/40	29.V.2008	300	49° 10’ N	07° 04’ E	*aurinia*
DE3	DE	Erkner	26/0	03.VI.2008	50	52° 25’ N	13° 45’ E	*aurinia*
EE1	EE	Salevere	25/29	05.VI.2008	20	58° 40’ N	23° 36’ E	*aurinia*
EE2	EE	Pühaküla	0/15	07.VI.2008	50	59° 21’ N	24° 31’ E	*aurinia*
ES1	ES	Bohonal de Ibor	0/41	28.V.2007	350	39° 47’ N	05° 28’ W	*beckeri*
ES2	ES	Rascafría	25/25	29.V.2007	1150	40° 53’ N	03° 51’ W	*beckeri*
ES3	ES	Santa Cruz de la Seros	0/40	30.V.2007	700	42° 33’ N	00° 37’ W	*beckeri*
ES4	ES	Sallent	24/45	05.VI.2007	350	41° 48’ N	01° 54’ E	*beckeri*
FR1	FR	Plan d’Alps	24/29	13.V.1998	900	43° 42’ N	06° 35’ E	*provincialis*
FR2	FR	La Louvières	0/30	08.V.1998	400	43° 37’ N	05° 45’ E	*provincialis*
FR3	FR	Col de Vars	0/6	25.VI.2006	2100	44° 32’ N	06° 42’ E	*glaciegenita*
FR4	FR	St-Georges-de-Luzençon	0/15	22.V.1998	400	44° 03’ N	02° 59’ E	*aurinia*
FR5	FR	Bois de l’Assise	0/25	30.V.1999	650	46° 03’ N	03° 48’ E	*aurinia*
FR6	FR	Voudenay le Château	22/30	21.VI.1999	300	47° 06’ N	04° 23’ E	*aurinia*
FR7	FR	Héric	15/15	15.V.1998	50	47° 24’ N	01° 38’ W	*aurinia*
FR8	FR	Rozereuilles	0/30	13.V.1999	250	49° 06' N	06° 04' E	*aurinia*
FR9	FR	Osmoy-Saint-Valéry	0/25	24.V.1999	50	49° 47' N	01° 19' E	*aurinia*
FR10	FR	Forêt de Trélon	24/30	26.V.1999	200	50° 04' N	04° 07' E	*aurinia*
IT1	IT	Carpegna	0/5	05.VI.2009	800	43° 47' N	12° 20' E	*provincialis*
IT2	IT	Montelago	24/31	04.VI.2009	850	43° 07' N	12° 57' E	*provincialis*
IT3	IT	Posta	0/38	04.VI.2009	1000	42° 31' N	13° 03' E	*provincialis*
IT4	IT	Campo di Giove	24/29	02.VI.2009	1000	42° 00' N	14° 02' E	*provincialis*
PT1	PT	Aljezur	24/42	18.V.2007	200	37° 13’ N	08° 46’ W	*beckeri*
PT2	PT	Ficalho	0/39	08.V.2007	500	37° 58’ N	07° 17’ W	*beckeri*
PT3	PT	Manteigas	0/40	25.V.2007	1200	40° 24’ N	07° 33’ W	*beckeri*
PT4	PT	Granja Nova	25/40	26.V.2007	750	41° 01’ N	07° 42’ W	*beckeri*
RO1	RO	Sárdu	25/40	08.VI.2008	550	46° 53’ N	23° 21’ E	*aurinia*
RO2	RO	Stána	0/12	10.VI.2008	600	46° 50’ N	23° 07’ E	*aurinia*
SI1	SI	Nanos	25/44	28.V.2009	900	45° 49’ N	14° 01’ E	*aurinia*
SI2	SI	Slavnik	0/40	29.V.2009	750	45° 33’ N	13° 58’ E	*aurinia*
SI3	SI	Iljirska Bistrica 1	0/21	29.V.2009	800	45° 33’ N	14° 18’ E	*aurinia*
SI4	SI	Iljirska Bistrica 2	0/23	30.V.2009	900	45° 34’ N	14° 18’ E	*aurinia*
SW1	SE	Uppsala	14/16	23.VI.2008	100	59° 58’ N	17° 09’ E	*aurinia*
DP1*	PT	Monte Velho	0/30	15.V.2007	150	37° 22' N	08° 25' W	*E*. *desfontainii*

Code, country (CTY; following ICO 3166), location, number of individuals for each marker (N; mtDNA/allozymes), capture date and geographic data.

* *Euphydryas desfontainii* (outgroup).

Specific permission for this study were granted by: Instituto da Conservacao da Natureza Lissabon for Portugal, Nature Protection Agency of the Province of Extremadura, Nature Protection Agency of the Province of Aragon, Nature Protection Agency of the Province of Catalonia, Directory of the National Parc of Majella (Italy), Naturschutzbehörde des Landes Salzburg, Obere Naturschutzbehörde des Saarlandes, Ministère de l'Environnement (Paris). Several samples were provided by local collectors or were collected together with them. No rights on private land were violated.

### Laboratory procedures

Total genomic DNA was extracted from butterfly legs using an adapted Chelex-100 (Bio-Rad) protocol [39]. A partial fragment (626 bp) of the mitochondrial cytochrome c oxidase subunit I (COI) gene was amplified by PCR using the universal primers HCO2198 and LCO1490 [40]. This region of the COI gene is the most variable according to previously published work [41]. Amplifications were performed in 25 μl reactions containing 1x colorless GoTaq Flexi Buffer, 2.0 mM MgCl_2_, 0.2 mM of each dNTP, 0.4 μM of each primer, 1U Go*Taq* Flexi DNA polymerase (Promega) and 50 ng template DNA. PCR cycling conditions were as follows: initial denaturation of 5 min at 95°C; followed by 40 cycles of 1 min at 95°C, 1 min at 51°C, 1 min at 72°C; and a final extension step of 10 min at 72°C. PCR products were purified by ethanol/sodium acetate precipitation. Sequencing was performed with the primer LCO1490 on an ABI 3130xl automated sequencer (Applied Biosystems—CCMAR, Portugal). Sequences were checked, manually edited and aligned using Geneious ver. 5.4 (http://www.geneious.com/). All sequences were deposited in GenBank (Accession Numbers: KT896753—KT897244).

For allozyme electrophoresis of 1,307 *E*. *aurinia* individuals, the whole abdomen of each imago was homogenized with ultrasound in Pgm-buffer [42] and centrifuged 3 min at 10,000 g. We used cellulose acetate plates, applying standard protocols [43,44]. A total of 17 allozyme loci were analysed (S3 Table).

### Statistical analyses

Information on mtDNA haplotype diversity, nucleotide diversity and frequency of each haplotype was extracted using DnaSP 5.10 [45] and Arlequin 3.5 [46]. The best model of nucleotide substitution was determined using jModelTest [47]. According to the Akaike Information Criteria (AIC) [48], the Tamura-Nei (I = 0.9270) [49] model fitted the data best (AIC 3898.0546). This model was used in further analyses as appropriate. Haplotype networks based on COI mtDNA data to depict relationships among haplotypes were performed with NETWORK v4.6 ([50], available at fluxus-engineering.com). Median-joining networks [50] that contained all possible equally short trees were simplified by running the maximum parsimony calculation option to eliminate superfluous nodes and links [51]. Pairwise location estimates of genetic differentiation plus 95% bootstrapped confidence intervals (10,000 replicates) were estimated using Weir and Cockerham’s [52] *F*_ST_ estimator and Jost’s [53] *D*_est_. Calculations were executed in the Diversity R package [54].

Allozyme alleles were labelled according to their relative mobility during electrophoresis. We used G-stat [55] to compute allele frequencies and parameters of allozyme genetic diversity (i.e. mean number of alleles per locus *A*, expected heterozygosity *H*_e_, observed heterozygosity *H*_o_, total percentage of polymorphic loci *P*_tot_ and percentage of polymorphic loci with the most common allele not exceeding 95% *P*_95_). We calculated allozyme allelic richness (*A*_R_) with Fstat [56] to allow for the different sample sizes from different locations. Populations FR3 and IT1 were excluded from this analysis because they were represented by too few individuals (*N* < 7). Allozyme locus-by-locus analyses of molecular variance (Amova), hierarchical genetic variance analyses, test of Hardy-Weinberg equilibrium and linkage disequilibrium were performed with Arlequin 3.5 [46]. Nei´s standard genetic distances [57], neighbour-joining phenograms [58] and bootstraps based on 1,000 interactions were calculated with Phylip 3.5.c [59].

Spatial genetic structure was assessed using two different approaches. First, we used the Spatial Analysis of Shared Alleles (SAShA) method [60] to establish non-panmixia on both mtDNA and allozyme data sets. This method is based on the premise that if groups are evolving locally then the same alleles or haplotypes are expected to co-occur in the same location more often than expected by chance. Second, a bayesian clustering algorithm of population assignment implemented in the R package Geneland 2.0 [61] produced a map that consolidated genetic and geographic data. To determine the number of genetic clusters, independent runs were implemented using 1,000,000 MCMC iterations with a burn-in period of 100 and a thinning value of 1,000. The value of *K* was set from 1 to 21 clusters on a correlated frequency model. We inferred the number of clusters from the modal value of *K* with the highest posterior probability.

## Results

### Mitochondrial DNA

**Fig 2:**
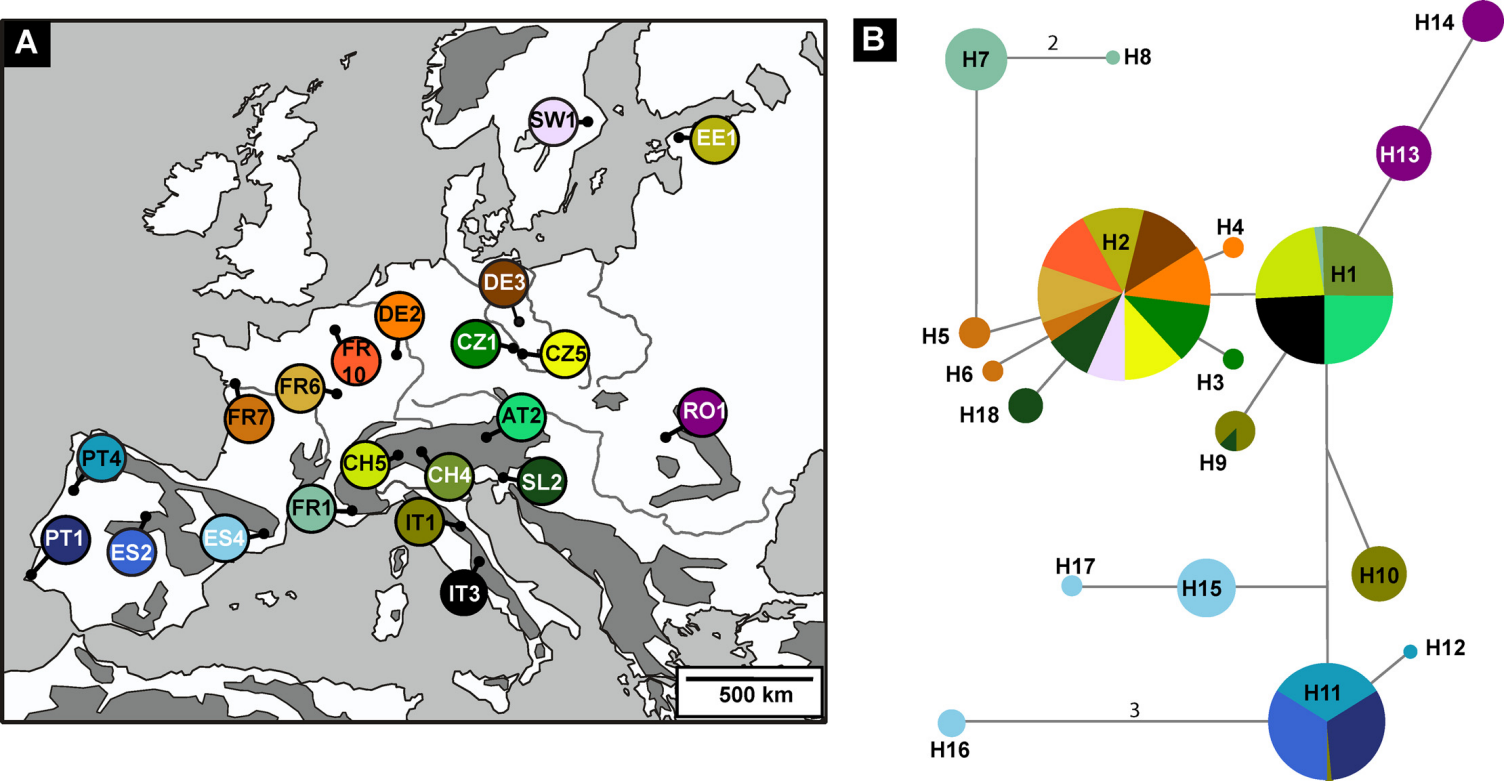
Median Joining Network indicating the relationship among COI haplotypes of *Euphydryas aurinia*. Each pie chart represents a different haplotype, made up of collection sites labelled by colour in which that haplotype occurs. Haplotypes connected by a line differ in sequence by one base pair unless otherwise indicated. The size of each pie chart is proportional to the relative frequency of the haplotype within the entire sample. The size of the collection site slices is influenced by both the frequency of the haplotype across the sites and the sample size for each site. For code abbreviations see Table 1.

We sequenced 492 individuals of *E*. *aurinia* from 21 sites in Europe for 626 bp of the COI gene. No pseudogenes were present as evidenced by the absence of stop codons, the prevalence of synonymous substitutions or low pairwise divergence. A total of 18 haplotypes were identified, including two non-synonymous and 17 parsimony informative sites (Fig 2). The limited numbers of nucleotide substitutions separating haplotypes suggest that the haplotypes are closely related and shared haplotypes were the most frequently detected haplotypes. Mean haplotype diversity was low (0.147 ± 0.038 SD) with haplotype numbers ranging from one to three in each location, and average nucleotide diversity was also low (0.038% ± 0.012% SD). Overall haplotype and nucleotide diversities were low (0.757 ± 0.014 SD and 0.34% ± 0.01% SD, respectively).

**Fig 3:**
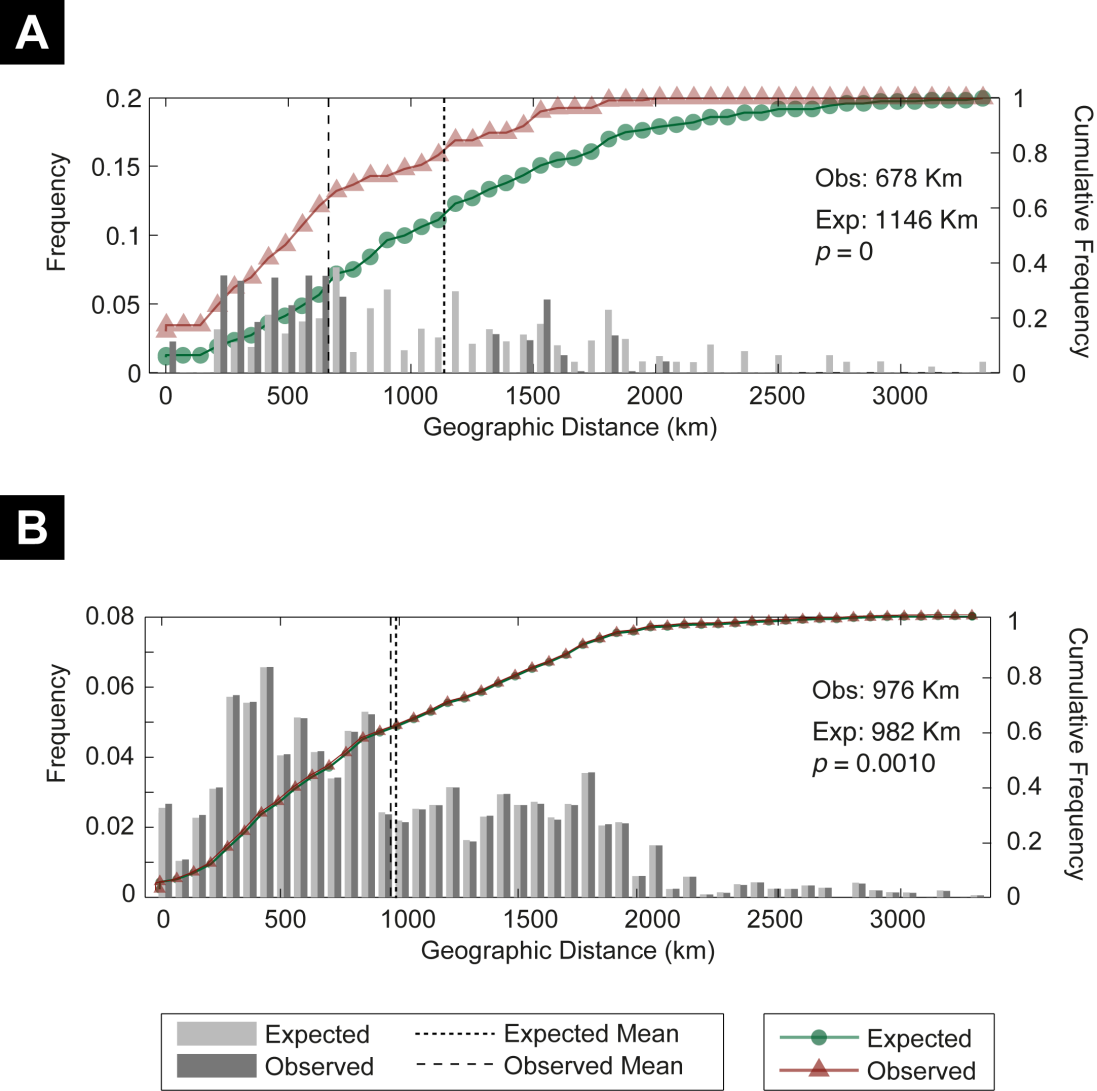
Spatial analysis of shared COI mtDNA haplotypes (A) and allozyme alleles (B) distribution of *Euphydryas aurinia*. Histograms represent the frequency of alleles between locations distance classes. Expected means and significance value were calculated with 1,000 randomized permutations of the data set. Vertical lines represent the mean of frequencies. Triangles and circles are the cumulative frequency of alleles at increasing distance. *P* value is the likelihood that the observed mean is greater than the expected.

The spatial analysis of shared alleles found that the arrangement of COI haplotypes was not equidistributed and was statistically different from that expected under panmixia (observed mean 678 km, expected mean 1,146 km, *p* < 0.0001) (Fig 3A). Pairwise differentiation was significant in 174 pairwise comparisons (92%) when measured using *D*_est_ and in 159 (84%) when using *F*_ST_ (S1 Table).

**Fig 4:**
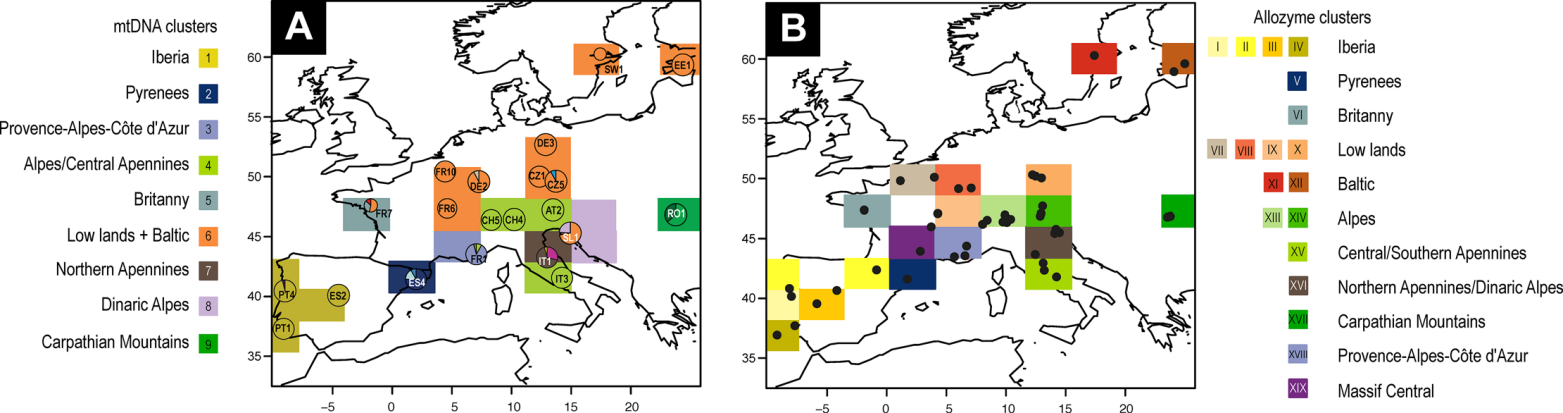
Spatial Bayesian clustering of *Euphydryas aurinia*. A. Mitochondrial DNA sequences, circles represent both locations and haplotypes found. B. Allozyme dataset, black dots represent locations. Spatial clustering suggests 19 distinct clusters across geographical regions.

Geneland analyses detected nine major clusters (mt1 –mt9) that correspond to Iberia (mt1), Pyrenees (mt2), Provence (mt3), Alps (including Central Apennines) (mt4), Brittany (mt5), Europe north of the Alps and the Pyrenees including Northern Europe (mt6), northern Apennines (mt7), Slovenia (mt8) and eastern Carpathian Basin (mt9) (Fig 4A). However, *D*_est_ and *F*_ST_ pairwise comparisons between clusters mt4 and mt6 were not significant (S2 Table).

### Allozymes

All 17 loci analysed were polymorphic and showed banding pattern consistent with known quaternary structures [43]. The polymorphisms in Fum were restricted to the sibling *E*. *desfontainii*, as all *E*. *aurinia* populations were monomorphic for this locus. The number of alleles per locus ranged from two (Mdh1) to ten (Pgi, Pgm, Pep_Phe-Pro_) (mean: 5.4 ± 2.7 SD). Allele frequencies can be obtained from the authors on personal request.

The mean number of alleles per locus (*A*) ranged from 1.41 to 2.53, with a mean of 2.00 (± 0.26 SD), while allelic richness (*A*_R_) ranged from 1.30 to 1.76 with a mean of 1.51 (± 0.10 SD) (Table 2). Excluding populations with respectively less than ten and 20 individuals yielded results between *A* and *A*_R_ (*A*_R10_ mean: 1.60 ± 0.11 SD; *A*_R20_ mean: 1.90 ± 0.17 SD). The highest values for allelic richness were found in populations from Provence, the western Alps and the Carpathian Basin. For the polymorphic loci with the most common allele not exceeding 95% (*P*_95_), frequencies ranged from 23.5% to 52.9%, mean: 42.1% (± 8.5% SD), while the total percentage of polymorphic loci (*P*_tot_) ranged from 35.3% to 82.4% with a mean of 57.9% (± 10.2% SD). The mean expected heterozygosity (*H*_e_) was 14.8% (± 2.7% SD), ranging from 9.1% to 22.9%, and the mean observed heterozygosity (*H*_o_) was estimated at 14.6% (± 2.6% SD), varying from 9.5% to 22.4%. The genetic diversity of the outgroup population *E*. *desfontainii* was considerably lower than the average of *E*. *aurinia*.

**Table 2:** Parameters of genetic diversity of allozymes analysed for 46 populations of *Euphydryas aurinia* and one population of *E*. *desfontainii* (DP1); sorting follows the twelve allozyme consensus groups al1 to al12.

Code	*H*_e_ (%)	*H*_o_ (%)	*P*_tot_ (%)	*P*_95_ (%)	*A*	*A*_R20_	*A*_R10_	*A*_R_	*N*	*N♀*	Allozyme groups	mtDNA groups
ES1	13.5	12.2	58.8	35.3	2.12	1.86	1.56	1.47	38.0	25	al1	
ES2	12.2	10.8	58.8	47.1	1.82	1.79	1.54	1.45	24.8	3	al1	mt1
FR4	13.4	12.8	47.1	41.2	(1.94)	-	1.63	1.52	13.9	0	al1	
PT1	14.4	16.0	47.1	29.4	1.82	1.67	1.50	1.45	37.7	16	al1	mt1
PT2	9.4	9.9	47.1	35.3	1.65	1.53	1.36	1.30	38.9	6	al1	
PT3	12.7	13.1	64.7	35.3	2.12	1.85	1.53	1.45	39.9	5	al1	
PT4	13.8	13.5	64.7	41.2	2.35	1.94	1.56	1.47	39.8	7	al1	mt1
ES3	14.4	15.0	52.9	41.2	1.71	1.57	1.43	1.39	39.1	11	al2	
ES4	14.7	13.9	47.1	41.2	1.94	1.71	1.48	1.42	39.8	16	al2	mt2
FR1	16.5	15.1	52.9	41.2	2.24	2.09	1.73	1.62	27.5	0	al3	mt3
FR2	16.7	15.3	58.8	47.1	2.24	2.06	1.68	1.58	28.6	0	al3	
CH5	16.2	17.2	64.7	41.2	2.06	2.00	1.68	1.57	22.8	5	al4	mt4
CH6	15.3	15.3	58.8	41.2	2.24	2.06	1.66	1.56	27.0	0	al4	
FR3	13.5	11.8	(41.2)	(41.2)	(1.53)	-	-	1.48	5.9	1	al4	
CH1	22.9	22.4	58.8	52.9	(2.06)	-	1.87	1.76	12.7	5	al5	
CH2	18.0	18.6	64.7	52.9	2.18	2.05	1.74	1.63	26.9	2	al5	
CH3	18.8	16.8	64.7	52.9	2.24	2.03	1.73	1.63	36.0	0	al5	
CH4	18.7	20.5	70.6	52.9	2.41	2.13	1.74	1.63	34.7	3	al5	mt4
AT2	16.4	16.9	52.9	47.1	2.00	1.84	1.63	1.55	37.0	1	al6	mt4
AT3	18.4	17.4	58.8	47.1	2.00	1.92	1.68	1.60	25.0	3	al6	
CZ1	17.2	16.5	70.6	52.9	2.18	2.12	1.76	1.63	25.0	0	al7	mt6
DE1	16.0	14.6	70.6	47.1	2.00	1.83	1.57	1.49	32.5	7	al7	
DE2	14.3	14.8	82.4	52.9	2.53	2.06	1.61	1.50	39.1	13	al7	mt6
DE3												mt6
FR5	13.2	13.1	58.8	47.1	2.00	1.91	1.55	1.45	24.2	0	al7	
FR6	10.5	10.5	47.1	23.5	1.59	1.57	1.38	1.33	21.5	0	al7	mt6
FR7	13.8	13.5	41.2	35.3	(1.71)	-	1.53	1.46	14.6	0	al7	mt5
FR8	13.8	12.7	58.8	41.2	2.18	2.03	1.64	1.53	29.8	0	al7	
FR9	9.7	11.1	58.8	23.5	1.88	1.75	1.40	1.33	24.8	0	al7	
FR10	14.1	14.4	47.1	47.1	1.77	1.69	1.51	1.44	29.9	0	al7	mt6
AT1	17.5	16.2	64.7	41.2	2.00	1.95	1.70	1.61	22.9	3	al8	
CZ2	13.6	14.7	70.6	52.9	1.94	1.88	1.61	1.51	24.0	0	al8	
CZ3	15.6	16.1	64.7	52.9	1.82	1.80	1.60	1.52	23.0	0	al8	
CZ4	16.5	16.7	64.7	52.9	2.18	2.07	1.73	1.62	25.0	0	al8	
CZ5												mt6
SW1	12.9	14.0	35.3	29.4	(1.53)	-	1.44	1.39	16.0	1	al8	mt6
SI1	15.0	13.6	64.7	47.1	2.41	2.08	1.69	1.57	44.0	2	al9	mt8
SI2	14.1	15.1	64.7	47.1	2.18	1.93	1.61	1.51	38.3	18	al9	
SI3	13.7	12.4	64.7	41.2	2.18	2.13	1.67	1.55	20.8	9	al9	
SI4	9.1	9.5	35.3	23.5	1.77	1.72	1.46	1.38	22.9	6	al9	
RO1	19.6	17.7	76.5	47.1	2.35	2.04	1.72	1.63	39.8	0	al10	mt9
RO2	19.0	18.6	52.9	41.2	(2.00)	-	1.77	1.65	11.9	1	al10	
IT1	13.1	15.3	(35.3)	(35.3)	(1.41)	-	-	1.41	5.0	0	al11	mt7
IT2	13.2	12.4	52.9	41.2	2.12	1.96	1.62	1.52	30.9	4	al11	
IT3	14.2	13.4	52.9	29.4	2.24	1.94	1.62	1.53	36.6	14	al11	mt4
IT4	12.4	12.5	47.1	35.3	1.94	1.87	1.59	1.50	28.3	14	al11	
EE1	15.0	14.6	58.8	35.3	1.77	1.72	1.56	1.49	28.3	7	al12	mt6
EE2	14.4	14.1	47.1	41.2	(1.65)	-	1.52	1.44	11.4	5	al12	
mean	14.8	14.6	57.9	42.1	2.06	1.90	1.60	1.51	27.5			
± SD	2.7	2.6	10.2	8.5	0.23	0.17	0.11	0.10	9.8			
DP1	8.5	9.2	41.2	41.2	1.53	1.49	1.42	1.35	30.0			

Expected heterozygosity (*H*_e_), observed heterozygosity (*H*_o_), total percentage of polymorphic loci (*P*_tot_), percentage of polymorphic loci with the most common allele not exceeding 95% (*P*_95_), mean number of alleles per locus (*A*), allelic richness for populations with number of individuals ≥20 (*A*_R20_), allelic richness for populations with individual numbers ≥10 (*A*_R10_), allelic richness for all populations (*A*_*R*_) and mean number of analysed individuals per locus (*N*). N♀ gives the number of females analysed for allozymes. Values excluded for the calculations of means (due to insufficient number of analysed individuals) are given in parentheses. For code abbreviations see Table 1.

None of the loci/population combinations showed any significant deviation from Hardy-Weinberg equilibrium, after Benjamini-Hochberg procedure for multiple testing [62,63]. Therefore, we performed further analyses using standard population genetic approaches.

**Fig 5:**
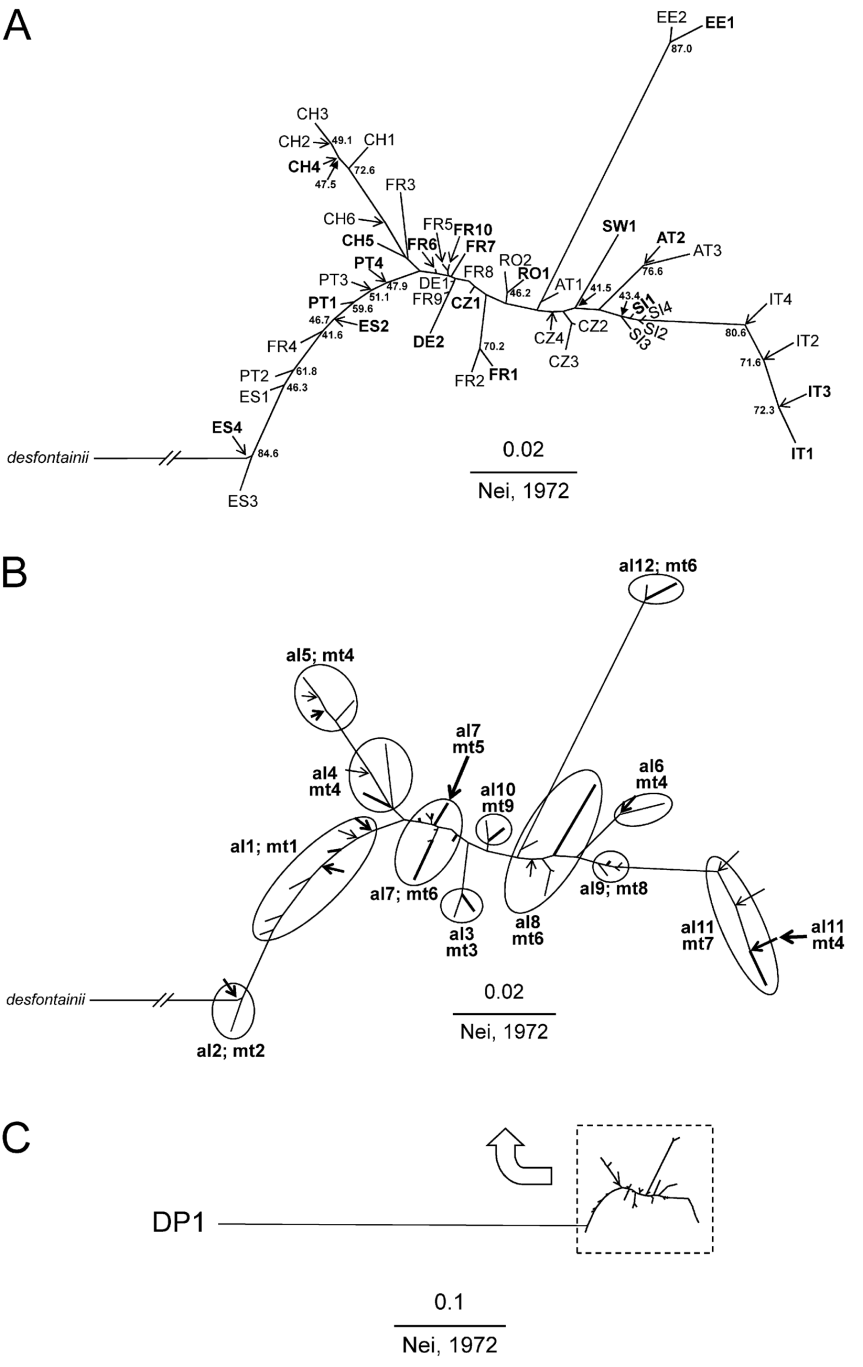
Neighbour-joining dendrogram of 46 populations of *Euphydryas aurinia* and one population of *E*. *desfontainii* based on genetic distances [57]. **A. Dendrogram with bootstrap values >40. For population codes see Table 1. B. Differentiation into twelve groups within the *E*. *aurinia* cluster (supported by bootstrap values and mtDNA patterns revealed by Geneland). The different group names are indicated for the allozyme groups and the mitochondrial groups. Populations otherwise not visible in the phenogram are indicated by arrows. C. Genetic distance among populations of *E*. *aurinia* and *E*. *desfontainii* (DP1; outgroup).** Populations used for mtDNA sequencing are given in bold in A and B.

The total genetic variance of all 46 *E*. *aurinia* populations was 1.585 with 0.326 genetic variance among populations (*F*_ST_ = 0.206; *p* < 0.001) and 0.020 genetic variance among individuals within populations (*F*_IS_ = 0.0158; *p* < 0.005) (Table 3). Excluding populations with less than 10 individuals did not change this result. The unbiased genetic distances [57] among all 46 samples ranged from 0.0021 to 0.1648 with a mean of 0.0502 (± 0.0300 SD). Based on these distances, a neighbour-joining dendrogam was created (Fig 5A; Fig 5C shows the rooting of the dendrogram with *E*. *desfontainii* as outgroup).

**Table 3:** Results of non-hierarchical variance analyses of allozyme data for different groups of *Euphydryas aurinia* and *E*. *desfontainii* in Europe.

Groups	*F*_ST_	*F*_IS_	Variance within individuals
*E*. *aurinia* + *E*. *desfontainii*	0.2518** (0.420)	0.0144* (0.018)	(1.229)
*E*. *aurinia*	0.2056** (0.326)	0.0158* (0.020)	(1.239)
Iberia (al1 + al2)	0.1043** (0.131)	0.0045 (0.005)	(1.123)
W Iberia (al1)	0.0704** (0.083)	0.0037 (0.004)	(1.086)
NE Iberia (al2)	0.0342* (0.044)	0.0067 (0.008)	(1.229)
Provence (al3)	0.0246* (0.036)	0.0876* (0.124)	(1.291)
Alps (al4—al6)	0.1199** (0.205)	-0.0001 (0.000)	(1.501)
W Alps (al4 + al5)	0.0528** (0.084)	0.0000 (-0.005)	(1.521)
SW Alps (al4)	0.0395** (0.054)	-0.0109 (-0014)	(1.329)
W Central Alps (al5)	0.0094 (0.015)	0.0000 (0.000)	(1.617)
E Alps (al6)	0.0199 (0.030)	0.0001 (0.012)	(1.452)
Western + Central Europe (al7—al10)	0.0897** (0.126)	0.0115 (0.015)	(1.265)
Western Europe (al7)	0.0375** (0.046)	0.0153 (0.018)	(1.155)
Central Europe (al8)	0.0543** (0.075)	-0.0181 (-0.024)	(1.330)
Slovenia (al9)	0.0175* (0.020)	0.0233 (0.027)	(1.118)
Carpathian Basin (al10)	0.0030 (0.005)	0.0842* (0.140)	(1.519)
Italy (al11)	0.0488** (0.058)	0.0355 (0.040)	(1.094)
Estonia (al12)	0.0246 (0.032)	0.0228 (0.029)	(1.233)

*F* value and variance component (in parentheses); variance within individuals only refers to variance component.

* *p* ≤ 0.05

** *p* ≤ 0.001; remaining values not significant.

The spatial analysis of shared alleles found that the arrangement of allozyme alleles was not equidistributed and was statistically different from the expectation under panmixia (observed mean 976 km, expected mean 982 km, *p* = 0.001) (Fig 3B). However, this difference although being highly significant was rather limited.

Information obtained from mtDNA and allozyme allele frequencies are geographically mostly consistent and in combination supported the existence of twelve genetic groups within *E*. *aurinia* (Fig 5B): western Iberia including SW France (i.e. FR4) (al1), Pyrenees and adjoining NE Iberia (al2), Provence (al3), SW Alps (al4), western Central Alps (al5), eastern Alps (al6), western Europe (al7), Central Europe (al8), Slovenia (al9), Carpathian Basin (al10), Italy (al11) and Estonia (al12). The mean genetic diversities of these twelve groups are given in Table 4. The samples of each of these twelve groups belong to one recognised subspecies; *E*. *aurinia beckeri*: groups al1 and al2; *E*. *aurinia provincialis*: groups al3 and al11; *E*. *aurinia glaciegenita*: groups al4, al 5 and al6; *E*. *aurinia aurini*a: all other groups. The only exception is population FR4 which is within the range of *E*. *aurinia aurinia*, in the western Massif Central, France, but is grouped with *E*. *aurinia beckeri* populations in group al1.

**Table 4:** Means of the analysed genetic diversity parameters of the twelve consensus groups of *Euphydryas aurinia* (al1 to al12) and the outgroup *E*. *desfontainii*.

Group	*H*_e_ (%)	*H*_o_ (%)	*P*_tot_ (%)	*P*_95_ (%)	*A*	*A*_R20_	*A*_R10_	*A*_*R*_	*N*	Allozyme group	mtDNA group
Western Iberia	12.8 (±1.6)	12.6 (±2.0)	55.5 (±8.2)	37.8 (±5.8)	1.98 (±0.26)	1.77 (±0.15)	1.53 (±0.08)	1.44 (±0.07)	33.3 (±10.1)	al1	mt1
NE Iberia	14.6 (±0.2)	14.5 (±0.8)	50.0 (±4.1)	41.2 (±0.0)	1.83 (±0.16)	1.64 (±0.10)	1.46 (±0.10)	1.41 (±0.02)	39.5 (±0.5)	al2	mt2
Provence	16.6 (±0.14)	15.2 (±0.14)	55.9 (±4.2)	44.2 (±4.2)	2.24 (±0.00)	2.08 (±0.02)	1.71 (±0.04)	1.60 (±0.03)	28.1 (±0.8)	al3	mt3
SW Alps	15.0 (±1.4)	14.8 (±2.7)	61.8 (±4.2)	41.2 (±0.0)	2.15 (±0.13)	2.03 (±0.04)	1.67 (±0.01)	1.54 (±0.05)	18.6 (±11.2)	al4	mt4
W Central Alps	19.6 (±2.23)	19.6 (±2.4)	64.7 (±4.8)	52.9 (±0.00)	2.28 (±0.12)	2.07 (±0.05)	1.77 (±0.07)	1.66 (±0.07)	27.6 (±10.7)	al5	mt4
Eastern Alps	17.4 (±1.4)	17.2 (±0.4)	55.9 (±4.2)	47.1 (±0.0)	2.00 (±0.00)	1.88 (±0.06)	1.66 (±0.04)	1.58 (±0.04)	31.0 (±8.5)	al6	mt4
Western Europe	13.6 (±2.4)	13.5 (±1.9)	59.5 (±13.3)	41.2 (±11.4)	2.02 (±0.29)	1.87 (±0.19)	1.55 (±0.12)	1.46 (±0.09)	26.8 (±7.0)	al7	mt5, mt6
Central Europe	15.2 (±1.9)	15.5 (±1.1)	60.0 (±14.0)	45.9 (±10.5)	1.99 (±0.15)	1.93 (±0.11)	1.62 (±0.11)	1.53 (±0.09)	22.2 (±3.6)	al8	mt6
Slovenia	13.0 (±2.6)	12.7 (±2.4)	57.4 (±14.7)	39.7 (±11.2)	2.14 (±0.27)	1.97 (±0.18)	1.61 (±0.10)	1.50 (±0.09)	31.5 (±11.4)	al9	mt8
Carpathian Basin	19.3 (±0.4)	18.2 (±0.6)	64.7 (±16.7)	44.2 (±4.2)	2.35	2.04	1.75 (±0.04)	1.64 (±0.01)	25.9 (±19.7)	al10	mt9
Italy	13.2 (±0.7)	13.4 (±1.3)	51.0 (±3.4)	35.3 (±5.9)	2.10 (±0.15)	1.92 (±0.05)	1.61 (±0.02)	1.49 (±0.05)	25.2 (±13.9)	al11	mt4, mt7
Estonia	14.7 (±0.4)	14.4 (±0.4)	53.0 (±8.3)	38.3 (±4.2)	1.77	1.72	1.54 (±0.03)	1.47 (±0.04)	19.9 (±12.0)	al12	mt6
All	14.8 (±2.7)	14.6 (±2.6)	57.9 (±10.2)	42.1 (±8.5)	2.06 (±0.23)	1.90 (±0.17)	1.60 (±0.11)	1.51 (±0.10)	27.5 (±9.8)	al1-12	mt1-9
*desfontainii*	8.5	9.2	41.2	41.2	1.53	1.49	1.42	1.35	30.0		

Abbreviations see Table 2.

Hierarchical variance analyses of allozyme data strongly confirm these twelve genetic groups (variance among groups: 0.304; *F*_CT_ = 0.1888; *p* < 0.001; variance within groups: 0.049; *F*_SC_ = 0.0378; *p* < 0.001; Table 5). Furthermore, subsequent hierarchical variance analyses based on regionalised parts of the entire data set (Table 5) supported the obtained structure among groups in the neighbour joining tree. Note that the tests presented here were mostly restricted to geographically neighbouring groups, to find whether patterns are supported or should be rejected. *F*_ST_ values within these groups (Table 3) are consistent with the genetic distances [57] within them. The Bayesian clustering of allozymes with GENELAND even distinguished 19 groups (best *K* = 19) within *E*. *aurinia* (Fig 4B); these groups were mostly congruent with the twelve groups outlined above as consensus between mtDNA and allozyme data, but revealed an even more fine-grained geographic pattern, which might however be biogeographically of limited significance in some of these cases.

**Table 5:** Results of hierarchical variance analyses of allozyme data among different groups of *Euphydryas aurinia* and *E*. *desfontainii* in Europe.

Groups	*F*_CT_	*F*_SC_	*F*_IS_	Variance within individuals
*E*. *aurinia* vs. *E*. *desfontainii*	0.5621** (2.019)	0.2074** (0.326)	0.0144* (0.018)	(1.229)
12 groups of *E*. *aurinia*	0.1888** (0.304)	0.0378** (0.049)	0.0158* (0.020)	(1.239)
Western + Central Europe (al7—al10) vs. Estonia (al12)	0.2251** (0.404)	0.1061** (0.147)	0.0178 (0.022)	(1.221)
Western Europe (al7) vs. Central Europe (al8) vs. Slovenia (al9) vs. Carpathian Basin (al10)	0.1056** (0.1514)	0.0328** (0.042)	0.0174 (0.022)	(1.220)
W Alps (al4 + al5) vs. E Alps (al6)	0.1530** (0.285)	0.0475** (0.075)	-0.0001 (-0.001)	(1.501)
SW Alps (al4) vs. W Central Alps (al5)	0.0663** (0.109)	0.0169** (0.026)	0.0000 (-0.005)	(1.521)
Provence (al3) vs. Italy (al11)	0.1674* (0.258)	0.0398* (0.051)	0.0569* (0.070)	(1.163)

*F* value and variance component (in parentheses); variance within individuals only refers to the variance component.

* *p* ≤ 0.05

** *p* ≤ 0.001; remaining values not significant.

Within the twelve consensus groups, the western Iberian cluster (al1) showed the strongest differentiation among populations (variance among populations: 0.083; *F*_ST_ = 0.0704; *p* < 0.001; variance among individuals within populations: 0.0037; *F*_IS_: 0.037; n.s.; Table 3). Also the Central European group (al8) (*F*_ST_ = 0.0543, *p* < 0.001) and the Italian group (al11) (*F*_ST_ = 0.0488, *p* < 0.001) showed remarkable within-group differentiation, while five other groups had low *F*_ST_ values ranging from not significant to 0.0246 (Table 3).

Hierarchical variance analyses showed a strong differentiation between the samples of *E*. *aurinia* and *E*. *desfontainii* (variance among groups: 0.02019; *F*_CT_ = 0.562; *p* < 0.001; variance within groups: 0.00326; *F*_SC_ = 0.207; *p* < 0.001; Table 5). Three taxon-specific alleles within the population of *E*. *desfontanii* were observed (Idh1: Allele 7; Fum: Allele 1 and 3). The mean genetic distance [57] between both taxa amounted 0.397 (± 0.080 SD).

## Discussion

The combination of the results from both allozymes and mtDNA supports the existence of twelve genetic groups. Different groups indicate different core areas in these regions; these are located in Iberia (2), Italy, Provence, Alps (3), Slovenia, Carpathian Basin, lowlands of West and Central Europe as well as Estonia, often with considerable additional substructures. However, despite the mostly consistent genetic pattern found between allozymes and mtDNA, differences were detected in peninsular Italy, the Alps, West and Central Europe as well as Estonia. In spite of the wide geographic sampling undertaken in the present study, the main caveat of this publication is the lack of samples from the Balkan Peninsula. This prevents us clarifying the importance for *E*. *aurinia* of the Ponto-Mediterranean refugium with its putative substructures (cf. [2]). Furthermore, the precise distribution of groups and detailed information on contact zones between them are not available for all twelve genetic groups.

### Genetic diversity

The *F*_ST_ values obtained here for *E*. *aurinia* are higher than those given previously for this species in nationwide surveys [64,65]. However our low value for the Provence area (*F*_ST_ = 0.0246) is much lower than that given in a previous study of this area with more samples (*F*_ST_ = 0.113 [65]). The parameters of genetic diversity (mtDNA and allozymes) of the *E*. *aurinia* populations are similar to many other representatives of the Nymphalidae (e.g. [66–70]) and exceed the values for most relict species or species with restricted distributions (e.g. [66,71,72]). However, the genetic diversity of *E*. *aurinia* was lower than in some very common Satyrinae [73–75] or Lycaenidae [70,76,77] and is also lower than in the North American mountain butterfly *Parnassius smintheus* [78] and in the mountain endemic *Erebia palarica* [71]. In general, the genetic diversity of *E*. *aurinia* thus matches the values of many moderately common and widespread butterfly species. The strong decline of *E*. *aurinia* populations in West and Central Europe during the last decades has so far not led to a remarkable loss of genetic diversity in a pan-European context.

### Genetic differentiation and biogeography

A spatially explicit Bayesian clustering method (Fig 4B) detecting small amounts of genetic differentiation [79] supported 19 allozyme clades. However, just twelve groups (joining several of these 19 clades) can be seen as biogeographically informative as a consensus between nuclear and mitochondrial genetic information. This structure of twelve groups is well reflected in the neighbour joining analysis of the allozyme polymorphisms (Fig 5B). However, two major discordances (central Italy, Estonia) exist between these two marker systems; these are discussed in detail below. The degree of genetic differentiation among the twelve distinct consensus groups of *E*. *aurinia* (Fig 5B), the values of hierarchical variance analyses and the differentiation at the mtDNA level are typical of butterfly species with strong intraspecific genetic structures [67,69,77,80–82]).

The clustering of the studied samples strongly suggests that recognised subspecies do not correspond to phylogenetic groups. *E*. *aurinia provincialis* should be split between its French populations (group al3) and its Italian populations (group al11). The name *E*. *aurinia aurunca* Turati, 1910 [29] may apply to the latter. Similarly, groups al4, al5 and al6 would all be in subspecies *E*. *aurinia glaciegenita* according to their distribution, while the clustering shows that al4 and al5 together belong to a different cluster than al6, which positions itself within *E*. *aurinia aurinia* groups; subspecies *E*. *aurinia glaciegenita* therefore does not correspond to a single cluster. Its eastern component, group al6 from high altitude Austria, may have had genetic exchange with lower altitude populations, without substantial changes in its phenotype (i.e. small size and dark colouring) and ecological requirements (i.e. it feeds on *Gentiana*), which are under strong selection in Alpine habitats. Our results clearly show that the Alpine populations sometimes known as *Euphydryas glaciegenita* belong to *Euphydryas aurinia*, and should not be given specific status [30].

The high degree of genetic differentiation of *E*. *aurinia* within Europe argues for the existence of several refugia during the last glaciation, if not before this. A similar assumption was made by Varga [83] who considered *E*. *aurinia* a polycentric species with holo-Palaearctic distribution. However, this author provided no details about the number and locations of refugia.

**Fig 6:**
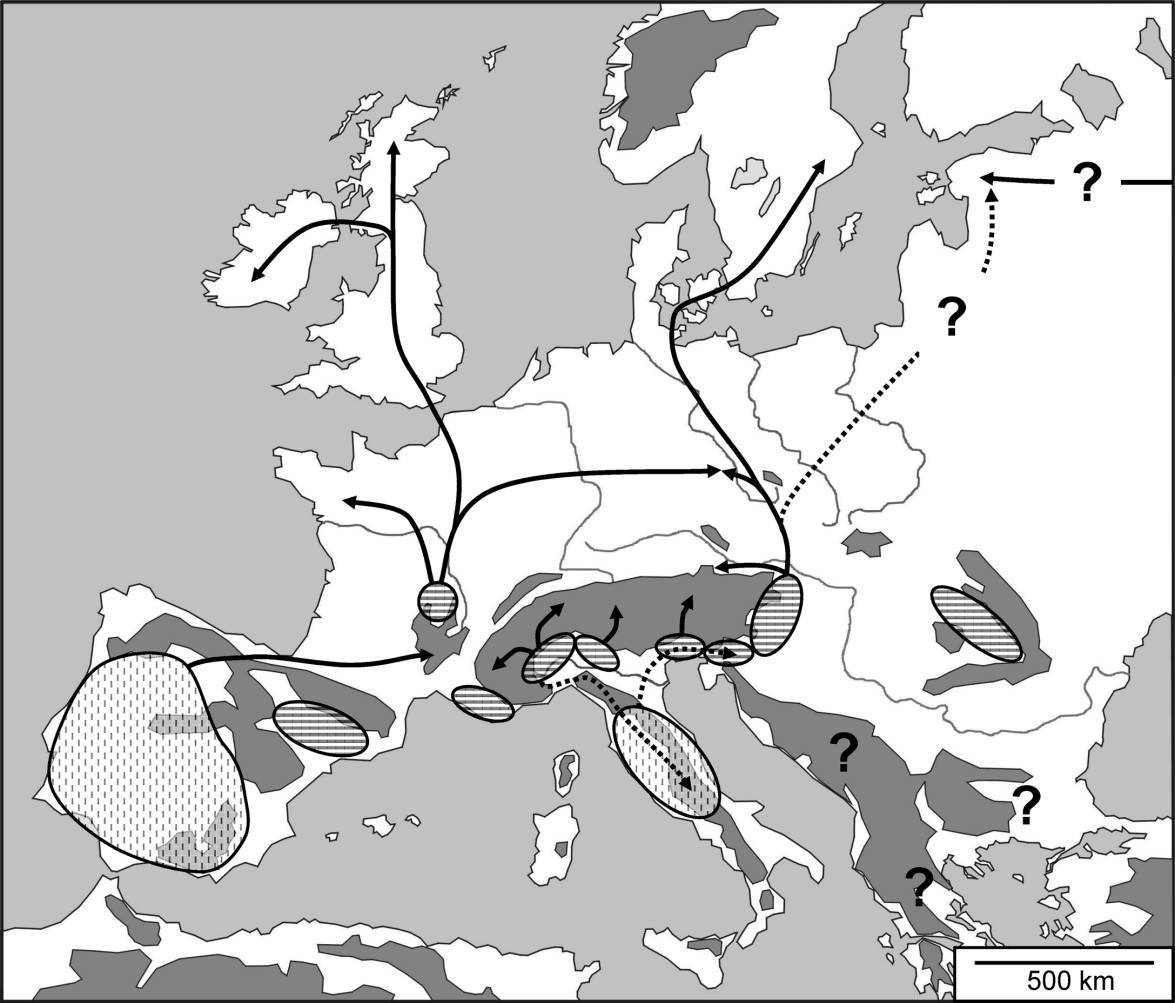
Hypothetical last glacial distribution patterns and postglacial expansion routes of *Euphydryas aurinia* in Europe. Striped pattern: putative continuous refugia; dashed pattern: putative structured refugia; solid arrows: proposed postglacial expansion routes; dashed arrows: supposed mtDNA introgression.

The three major Mediterranean peninsulas represented important refugia during the last ice age for many different animal and plant species [1,84–86] and might have been of importance also for *E*. *aurinia*. Our analyses revealed independent genetic groups in south-western Europe (al1, al2) and Italy (al11) which respectively indicate an atlanto-Mediterranean and an adriato-Mediterranean glacial refugium for this species. There might also be several subrefugia in Iberia and Central Italy (Fig 6). The genetic structure of the neighbour joining tree based on allozyme data and the mtDNA haplotype network support the survival of *E*. *aurinia* in at least two subrefugia in western Iberia (al1) and south of the Pyrenees (al2) (Fig 6) during the last ice age. Such an East-West discontinuity has repeatedly been observed for Iberia [87–91]. Further, more subtle regional substructuring of the western Iberian group (al1) during the last ice age is conceivable based on the remarkable allozyme structure throughout this region. Such refugia-within-refugia structures have been repeatedly demonstrated in Iberia for different taxonomic groups [92–96]. Additionally, it is likely that the remarkable ecological differentiation of Iberian *E*. *aurinia*, e.g. the larvae feeding on *Lonicera* species thus linking the species to hedge structures [19,20], and not *Succisa pratensis* and *Scabiosa columbaria* as in most of the other lowland populations [21–24], is the result of long-lasting allopatry, perhaps in combination with different selective pressures acting in the Iberian and the other refugia.

Similar events might have taken place in Central Italy (al11), although no clear conclusions can be drawn concerning the location of different subrefugia in this area because only four populations were analysed. However, polycentricity of *E*. *aurinia* in Italy is supported by phylogeographic structures of many other species showing often numerous genetic groups and hence core areas scattered over Italy [97–102].

Furthermore, the more southern Italian population IT3 shares its haplotype with populations from the Alps and has no haplotypes endemic to peninsular Italy, unlike the more northern population IT2. This discrepancy between nuclear and mitochondrial genes is most likely due to mitochondrial introgression from a more northern perialpine centre of survival (see below) along the Apennines to the South.

A ponto-Mediterranean refugium of *E*. *aurinia* could not currently be verified due to lack of samples. However, the descriptions of independent subspecies from the Balkans (*E*. *aurinia balcanica* Schawerda, 1908; *E*. *aurinia bulgarica* Fruhstorfer, 1916) are evidence for further centres of genetic differentiation in this area. Furthermore, refugia in the Balkan Peninsula are the rule for widespread species of Mediterranean origin [1–3], thus making it probable that this is also the case in *E*. *aurinia*.

Another Mediterranean glacial refugium in southern France seems to be most likely for *E*. *aurinia* due to the remarkable genetic differentiation of the populations from Provence for both genetic marker sets (al3, mt3) (Fig 6). The much higher than average genetic diversities of allozymes of the respective populations provide further support for a Provence refugium. Similar refugia in this area were assumed for e.g. *Zerynthia polyxena* [103], *Carabus auronitens* [104], *Natrix natrix* [102], *Vipera aspis* [105], *Quercus suber* [106] and *Fagus sylvatica* [17].

Beside these Mediterranean glacial refugia, the importance of extra-Mediterranean refugia has been revealed for many temperate European species (see [8] for a recent review). Such refugia might also be the most probable explanation for an independent western and eastern genetic group of *E*. *aurinia* in West and Central Europe. We suggest that the origin of the western group (al7) lies in an extra-Mediterranean refugium in Central France (Fig 6). The most probable location of this extra-Mediterranean refugium seems to be in the area north of the French Massif Central, a region with frequent indications of such refugia [8], from where postglacial expansion started towards the North and West of France as well as in an easterly direction towards western Bohemia (westernmost Czech Republic) (Fig 6). Additional analyses including samples from eastern Germany (e.g. Saxony, eastern Germany) might help to establish in more detail the contact zone between the western and eastern genetic group of *E*. *aurinia* in Central Europe.

The Central European group of *E*. *aurinia* (al8) might have had its origin east/south-east of the Alps, also an important area of extra-Mediterranean glacial refugia for many animal and plant species (e.g. [2,11,13,17,80,107,108]). Postglacial expansion from this refugium seems to run mainly towards the North and, at a limited scale, in a westerly direction (Fig 6). Hence, this genetic group of *E*. *aurinia* reached the southern parts of Sweden, while no populations of this group are known west of Salzburg (eastern Alps) and Bohemia (western Czech Republic). This refugium may have been in close contact with a Slovenian refugium, which might have been just a subrefugium of the former, in which stronger postglacial introgression processes led to its populations’ uniqueness.

Genetic differentiation at the nuclear (al10) and mitochondrial (mt9) level speaks for an independent extra-Mediterranean glacial refugium in the Carpathian Basin. The extraordinarily high genetic diversities of the here analysed populations from Transylvania support the high importance of this Basin as a centre for extra-Mediterranean survival with particularly favourable conditions [8], as also supported for several other species (e.g. [11–13,107]).

Expansions from south-eastern Europe up to the Baltic States (as e.g. in the brown bear [109] or the butterfly *Coenonympha arcania* [110]) seem unlikely due to the strong genetic differentiation of the Estonian populations at the nuclear level (al12), with several endemic allozyme alleles. In fact, the Baltic populations of *E*. *aurinia* might have their origin in the East (possibly in the southern Ural, cf. [111]). However, the mtDNA haplotype obtained from Estonia is not different from the ones of Central Europe. Therefore, we assume that mitochondrial introgression of the Central European group has substituted a putative former eastern haplotype, but did not (or only marginally) influence the nuclear genetic information.

All populations from the Alps belong to the distinct mtDNA group mt4. These Alpine populations consistently show a different phenotype, which is considerably smaller than all ordinary lowland populations, and the general colouring of the wings is much darker; furthermore, these individuals have a different mode of flight (much closer to the ground and also quicker), use different larval food plants (i.e. *Gentiana* species) and do not show protandry like lowland populations do [18,25,26,112]. These ecological shifts might have happened during isolation in perialpine refugia (ice ages) and in their non-refugial environments (always during interglacials). As most of these differences represent specific adaptations to survive the harsh conditions of high mountain ecosystems [26], they might be the result of directed selection and not of random drift.

At the nuclear level, however, our study also revealed remarkable genetic differentiation within the Alps (*F*_ST_ = 0.1199), so that three Alpine genetic groups can be distinguished (al4, al5, al6) (Tables 3 and 5; Fig 5). This calls for the existence of not just one but several perialpine glacial refugia, at least during the last ice age. The two genetic groups in the western Alps (al4, al5) show moderate genetic differentiation from each other (*F*_CT_ = 0.0663), indicating that both groups arose by allopatric differentiation during presumably one ice age. This relatively short time of divergence accords with the lack of mtDNA differentiation among the Alpine populations. The respective refugia might have been located near to the foot of the Cottian Alps (al4) and in the area south of the Upper Italian lakes (al5) (Fig 6). Especially the refugium of the western Central Alps group (al5) might have been of particular importance, as underlined by the rather high allozyme diversity of the populations analysed from this group. Evidence for similar refugia in these areas is not only found in various Alpine plant species [113–120], but also the beetle *Oreina elongata* [121].

The populations of *E*. *aurinia* in the Hohe Tauern (eastern Alps, al6) show strong genetic differentiation from those of the western and central Alps (*F*_CT_ = 0.1530). This result indicates a longer isolation of the populations of the eastern Alps (but note the missing differentiation at the mtDNA level) and an independent refugium in this region. Especially the area south of the Carnian and Julian Alps apparently offered suitable environmental conditions for many species, often resulting in higher genetic diversities of the genetic groups in the eastern Alps [10,82,122–124]. We therefore assume that the mentioned region also represented an important glacial refugium for *E*. *aurinia*. Postglacial expansion might have been mainly in a northern direction into the higher Alps, as in the Slovenian populations (al9), but with the latter showing some introgression from the eastern Alps and the Apennines group at the mtDNA level.

## Conclusion

*Euphydryas aurinia* shows a large variety of morphological as well as ecological adaptations within Europe, which have been the cause of continuous taxonomic debate. However, the results of the present study revealed that the ecological adaptations are most likely the result of strong intraspecific differentiation processes and not a result of the existence of a super-species complex. Hence, *E*. *aurinia* represents a rare model organism that combines attributes of Mediterranean, continental as well as alpine faunal elements within one single species. The complex phylogeography of *E*. *aurinia* might therefore be an example of more complicated patterns of differentiation in species showing adaptations to various climatic and altitudinal conditions, as well as host plants, across the western Palaearctic.

## Supporting Information

S1 Table Pairwise-location *D*_est_ (estimated) and *F*_ST_ values for mtDNA sequences *Euphydryas aurinia*. Site abbreviations defined in Table 1.
(PDF)

S2 Table Pairwise-group *D*_est_ (estimated) and *F*_ST_ values for mtDNA sequences *Euphydryas aurinia*. Groups defined in Table 2.
(PDF)

S3 Table Electrophoresis conditions for the different enzymes analysed for *Euphydryas aurini*a. (PDF)
