# Supplementary material for: Three in One—Multiple Faunal Elements within an Endangered European Butterfly Species
Source: PLoS One. 2015 Nov 13;10(11):e0142282. doi: 10.1371/journal.pone.0142282 (PMC4643965; doi:10.1371/journal.pone.0142282)
Supplement: S1 Table — Site abbreviations defined in Table 1. (PDF) [file pone.0142282.s001.pdf]

Table S1.

Pairwise-location *Dest* (estimated) and *Fst* values for mtDNA sequences *E. aurinia*. Site abbreviations defined in Table 1.
(significance obtained by 10000 bootstraps)

D jost

Pairs of locations	*Dest*	Lower 95%CI	Upper 95%CI	Significance
AT2 vs. CZ1	1.000	1.000	1.000	sig.
AT2 vs. CZ5	1.000	1.000	1.000	sig.
AT2 vs. DE2	1.000	1.000	1.000	sig.
AT2 vs. DE3	1.000	1.000	1.000	sig.
AT2 vs. EE1	1.000	1.000	1.000	sig.
AT2 vs. ES2	1.000	1.000	1.000	sig.
AT2 vs. ES4	1.000	1.000	1.000	sig.
AT2 vs. FR1	0.942	0.851	1.002	sig.
AT2 vs. FR6	1.000	1.000	1.000	sig.
AT2 vs. FR7	1.000	1.000	1.000	sig.
AT2 vs. CH4	0.000	0.000	0.000	sig.
AT2 vs. CH5	0.000	0.000	0.000	sig.
AT2 vs. IT1	1.000	1.000	1.000	sig.
AT2 vs. IT3	0.000	0.000	0.000	sig.
AT2 vs. PT1	1.000	1.000	1.000	sig.
AT2 vs. PT4	1.000	1.000	1.000	sig.
AT2 vs. RO1	1.000	1.000	1.000	sig.
AT2 vs. SLO	1.000	1.000	1.000	sig.
AT2 vs. SW1	1.000	1.000	1.000	sig.
CZ1 vs. CZ5	0.005	-0.004	0.040	n.s.
CZ1 vs. DE2	0.004	-0.007	0.045	n.s.
CZ1 vs. DE3	0.005	-0.004	0.040	n.s.
CZ1 vs. EE1	0.005	-0.004	0.040	n.s.
CZ1 vs. ES2	1.000	1.000	1.000	sig.
CZ1 vs. ES4	1.000	1.000	1.000	sig.
CZ1 vs. FR1	0.284	0.118	0.489	sig.
CZ1 vs. FR6	0.005	-0.004	0.040	n.s.
CZ1 vs. FR7	0.212	0.000	0.573	sig.
CZ1 vs. CH4	1.000	1.000	1.000	sig.
CZ1 vs. CH5	1.000	1.000	1.000	sig.
CZ1 vs. IT1	1.000	1.000	1.000	sig.
CZ1 vs. IT3	1.000	1.000	1.000	sig.
CZ1 vs. PT1	1.000	1.000	1.000	sig.
CZ1 vs. PT4	1.000	1.000	1.000	sig.
CZ1 vs. RO1	1.000	1.000	1.000	sig.
CZ1 vs. SLO	0.066	-0.008	0.225	n.s.
CZ1 vs. SW1	0.005	-0.004	0.039	n.s.
CZ5 vs. DE2	0.005	-0.004	0.040	n.s.
CZ5 vs. DE3	0.000	0.000	0.000	sig.
CZ5 vs. EE1	0.000	0.000	0.000	sig.
CZ5 vs. ES2	1.000	1.000	1.000	sig.
CZ5 vs. ES4	1.000	1.000	1.000	sig.
CZ5 vs. FR1	0.303	0.138	0.500	sig.
CZ5 vs. FR6	0.000	0.000	0.000	sig.
CZ5 vs. FR7	0.237	0.026	0.593	sig.
CZ5 vs. CH4	1.000	1.000	1.000	sig.
CZ5 vs. CH5	1.000	1.000	1.000	sig.
CZ5 vs. IT1	1.000	1.000	1.000	sig.
CZ5 vs. IT3	1.000	1.000	1.000	sig.
CZ5 vs. PT1	1.000	1.000	1.000	sig.
CZ5 vs. PT4	1.000	1.000	1.000	sig.
CZ5 vs. RO1	1.000	1.000	1.000	sig.
CZ5 vs. SLO	0.082	0.001	0.239	sig.
CZ5 vs. SW1	0.000	0.000	0.000	sig.
DE2 vs. DE3	0.005	-0.004	0.040	n.s.
DE2 vs. EE1	0.005	-0.004	0.040	n.s.
DE2 vs. ES2	1.000	1.000	1.000	sig.
DE2 vs. ES4	1.000	1.000	1.000	sig.
DE2 vs. FR1	0.284	0.115	0.488	sig.
DE2 vs. FR6	0.005	-0.004	0.040	n.s.
DE2 vs. FR7	0.212	0.002	0.573	sig.
DE2 vs. CH4	1.000	1.000	1.000	sig.
DE2 vs. CH5	1.000	1.000	1.000	sig.
DE2 vs. IT1	1.000	1.000	1.000	sig.
DE2 vs. IT3	1.000	1.000	1.000	sig.
DE2 vs. PT1	1.000	1.000	1.000	sig.
DE2 vs. PT4	1.000	1.000	1.000	sig.
DE2 vs. RO1	1.000	1.000	1.000	sig.
DE2 vs. SLO	0.066	-0.008	0.225	n.s.
DE2 vs. SW1	0.005	-0.004	0.039	n.s.
DE3 vs. EE1	0.000	0.000	0.000	sig.
DE3 vs. ES2	1.000	1.000	1.000	sig.
DE3 vs. ES4	1.000	1.000	1.000	sig.
DE3 vs. FR1	0.303	0.139	0.500	sig.
DE3 vs. FR6	0.000	0.000	0.000	sig.
DE3 vs. FR7	0.237	0.026	0.593	sig.
DE3 vs. CH4	1.000	1.000	1.000	sig.
DE3 vs. CH5	1.000	1.000	1.000	sig.
DE3 vs. IT1	1.000	1.000	1.000	sig.
DE3 vs. IT3	1.000	1.000	1.000	sig.
DE3 vs. PT1	1.000	1.000	1.000	sig.
DE3 vs. PT4	1.000	1.000	1.000	sig.
DE3 vs. RO1	1.000	1.000	1.000	sig.
DE3 vs. SLO	0.082	0.001	0.239	sig.
DE3 vs. SW1	0.000	0.000	0.000	sig.
EE1 vs. ES2	1.000	1.000	1.000	sig.
EE1 vs. ES4	1.000	1.000	1.000	sig.
EE1 vs. FR1	0.303	0.139	0.500	sig.
EE1 vs. FR6	0.000	0.000	0.000	sig.
EE1 vs. FR7	0.237	0.026	0.593	sig.
EE1 vs. CH4	1.000	1.000	1.000	sig.
EE1 vs. CH5	1.000	1.000	1.000	sig.
EE1 vs. IT1	1.000	1.000	1.000	sig.
EE1 vs. IT3	1.000	1.000	1.000	sig.
EE1 vs. PT1	1.000	1.000	1.000	sig.

Pairs of locations	*Dest*	Lower 95%CI	Upper 95%CI	Significance
EE1 vs. PT4	1.000	1.000	1.000	sig.
EE1 vs. RO1	1.000	1.000	1.000	sig.
EE1 vs. SLO	0.082	0.001	0.239	sig.
EE1 vs. SW1	0.000	0.000	0.000	sig.
ES2 vs. ES4	1.000	1.000	1.000	sig.
ES2 vs. FR1	1.000	1.000	1.000	sig.
ES2 vs. FR6	1.000	1.000	1.000	sig.
ES2 vs. FR7	1.000	1.000	1.000	sig.
ES2 vs. CH4	1.000	1.000	1.000	sig.
ES2 vs. CH5	1.000	1.000	1.000	sig.
ES2 vs. IT1	0.945	0.822	1.000	sig.
ES2 vs. IT3	1.000	1.000	1.000	sig.
ES2 vs. PT1	0.000	0.000	0.000	sig.
ES2 vs. PT4	0.001	-0.002	0.012	n.s.
ES2 vs. RO1	1.000	1.000	1.000	sig.
ES2 vs. SLO	1.000	1.000	1.000	sig.
ES2 vs. SW1	1.000	1.000	1.000	sig.
ES4 vs. FR1	1.000	1.000	1.000	sig.
ES4 vs. FR6	1.000	1.000	1.000	sig.
ES4 vs. FR7	1.000	1.000	1.000	sig.
ES4 vs. CH4	1.000	1.000	1.000	sig.
ES4 vs. CH5	1.000	1.000	1.000	sig.
ES4 vs. IT1	1.000	1.000	1.000	sig.
ES4 vs. IT3	1.000	1.000	1.000	sig.
ES4 vs. PT1	1.000	1.000	1.000	sig.
ES4 vs. PT4	1.000	1.000	1.000	sig.
ES4 vs. RO1	1.000	1.000	1.000	sig.
ES4 vs. SLO	1.000	1.000	1.000	sig.
ES4 vs. SW1	1.000	1.000	1.000	sig.
FR1 vs. FR6	0.303	0.138	0.500	sig.
FR1 vs. FR7	0.359	0.127	0.664	sig.
FR1 vs. CH4	0.942	0.851	1.002	sig.
FR1 vs. CH5	0.942	0.851	1.002	sig.
FR1 vs. IT1	1.000	1.000	1.000	sig.
FR1 vs. IT3	0.942	0.851	1.002	sig.
FR1 vs. PT1	1.000	1.000	1.000	sig.
FR1 vs. PT4	1.000	1.000	1.000	sig.
FR1 vs. RO1	1.000	1.000	1.000	sig.
FR1 vs. SLO	0.284	0.111	0.509	sig.
FR1 vs. SW1	0.301	0.136	0.498	sig.
FR6 vs. FR7	0.236	0.026	0.593	sig.
FR6 vs. CH4	1.000	1.000	1.000	sig.
FR6 vs. CH5	1.000	1.000	1.000	sig.
FR6 vs. IT1	1.000	1.000	1.000	sig.
FR6 vs. IT3	1.000	1.000	1.000	sig.
FR6 vs. PT1	1.000	1.000	1.000	sig.
FR6 vs. PT4	1.000	1.000	1.000	sig.
FR6 vs. RO1	1.000	1.000	1.000	sig.
FR6 vs. SLO	0.082	0.001	0.239	sig.
FR6 vs. SW1	0.000	0.000	0.000	sig.
FR7 vs. CH4	1.000	1.000	1.000	sig.
FR7 vs. CH5	1.000	1.000	1.000	sig.
FR7 vs. IT1	1.000	1.000	1.000	sig.
FR7 vs. IT3	1.000	1.000	1.000	sig.
FR7 vs. PT1	1.000	1.000	1.000	sig.
FR7 vs. PT4	1.000	1.000	1.000	sig.
FR7 vs. RO1	1.000	1.000	1.000	sig.
FR7 vs. SLO	0.203	-0.001	0.552	n.s.
FR7 vs. SW1	0.234	0.024	0.592	sig.
CH4 vs. CH5	0.000	0.000	0.000	sig.
CH4 vs. IT1	1.000	1.000	1.000	sig.
CH4 vs. IT3	0.000	0.000	0.000	sig.
CH4 vs. PT1	1.000	1.000	1.000	sig.
CH4 vs. PT4	1.000	1.000	1.000	sig.
CH4 vs. RO1	1.000	1.000	1.000	sig.
CH4 vs. SLO	1.000	1.000	1.000	sig.
CH4 vs. SW1	1.000	1.000	1.000	sig.
CH5 vs. IT1	1.000	1.000	1.000	sig.
CH5 vs. IT3	0.000	0.000	0.000	sig.
CH5 vs. PT1	1.000	1.000	1.000	sig.
CH5 vs. PT4	1.000	1.000	1.000	sig.
CH5 vs. RO1	1.000	1.000	1.000	sig.
CH5 vs. SLO	1.000	1.000	1.000	sig.
CH5 vs. SW1	1.000	1.000	1.000	sig.
IT1 vs. IT3	1.000	1.000	1.000	sig.
IT1 vs. PT1	0.945	0.822	1.000	sig.
IT1 vs. PT4	0.945	0.820	1.000	sig.
IT1 vs. RO1	1.000	1.000	1.000	sig.
IT1 vs. SLO	0.979	0.916	1.000	sig.
IT1 vs. SW1	1.000	1.000	1.000	sig.
IT3 vs. PT1	1.000	1.000	1.000	sig.
IT3 vs. PT4	1.000	1.000	1.000	sig.
IT3 vs. RO1	1.000	1.000	1.000	sig.
IT3 vs. SLO	1.000	1.000	1.000	sig.
IT3 vs. SW1	1.000	1.000	1.000	sig.
PT1 vs. PT4	0.001	-0.002	0.012	n.s.
PT1 vs. RO1	1.000	1.000	1.000	sig.
PT1 vs. SLO	1.000	1.000	1.000	sig.
PT1 vs. SW1	1.000	1.000	1.000	sig.
PT4 vs. RO1	1.000	1.000	1.000	sig.
PT4 vs. SLO	1.000	1.000	1.000	sig.
PT4 vs. SW1	1.000	1.000	1.000	sig.
RO1 vs. SLO	1.000	1.000	1.000	sig.
RO1 vs. SW1	1.000	1.000	1.000	sig.
SLO vs. SW1	0.080	0.000	0.237	sig.

FST

Pairs of locations	*FST*	Lower 95%CI	Upper 95%CI	Significance
AT2 vs. CZ1	0.923	0.831	0.998	sig.
AT2 vs. CZ5	1.000	1.000	1.000	sig.
AT2 vs. DE2	0.923	0.830	0.996	sig.
AT2 vs. DE3	1.000	1.000	1.000	sig.
AT2 vs. EE1	1.000	1.000	1.000	sig.
AT2 vs. ES2	1.000	1.000	1.000	sig.
AT2 vs. ES4	0.794	0.700	0.912	sig.
AT2 vs. FR1	0.640	0.579	0.690	sig.
AT2 vs. FR6	1.000	1.000	1.000	sig.
AT2 vs. FR7	0.750	0.701	0.822	sig.
AT2 vs. CH4	-	-	-	-
AT2 vs. CH5	-	-	-	-
AT2 vs. IT1	0.760	0.697	0.847	sig.
AT2 vs. IT3	-	-	-	-
AT2 vs. PT1	1.000	1.000	1.000	sig.
AT2 vs. PT4	0.960	0.889	0.999	sig.
AT2 vs. RO1	0.760	0.729	0.849	sig.
AT2 vs. SLO	0.780	0.705	0.882	sig.
AT2 vs. SW1	1.000	1.000	1.000	sig.
CZ1 vs. CZ5	0.039	-0.012	0.152	n.s.
CZ1 vs. DE2	0.002	-0.049	0.096	n.s.
CZ1 vs. DE3	0.042	-0.010	0.156	n.s.
CZ1 vs. EE1	0.042	-0.010	0.156	n.s.
CZ1 vs. ES2	0.923	0.831	0.998	sig.
CZ1 vs. ES4	0.715	0.581	0.847	sig.
CZ1 vs. FR1	0.290	0.153	0.447	sig.
CZ1 vs. FR6	0.034	-0.015	0.144	n.s.
CZ1 vs. FR7	0.276	0.043	0.558	sig.
CZ1 vs. CH4	0.923	0.831	0.998	sig.
CZ1 vs. CH5	0.920	0.825	0.997	sig.
CZ1 vs. IT1	0.681	0.568	0.804	sig.
CZ1 vs. IT3	0.922	0.828	0.997	sig.
CZ1 vs. PT1	0.922	0.828	0.997	sig.
CZ1 vs. PT4	0.883	0.753	0.996	sig.
CZ1 vs. RO1	0.683	0.580	0.797	sig.
CZ1 vs. SLO	0.121	0.000	0.296	n.s.
CZ1 vs. SW1	0.009	-0.035	0.105	n.s.
CZ5 vs. DE2	0.039	-0.013	0.152	n.s.
CZ5 vs. DE3	-	-	-	-
CZ5 vs. EE1	-	-	-	-
CZ5 vs. ES2	1.000	1.000	1.000	sig.
CZ5 vs. ES4	0.790	0.695	0.910	sig.
CZ5 vs. FR1	0.355	0.221	0.507	sig.
CZ5 vs. FR6	-	-	-	-
CZ5 vs. FR7	0.403	0.184	0.648	sig.
CZ5 vs. CH4	1.000	1.000	1.000	sig.
CZ5 vs. CH5	1.000	1.000	1.000	sig.
CZ5 vs. IT1	0.755	0.692	0.844	sig.
CZ5 vs. IT3	1.000	1.000	1.000	sig.
CZ5 vs. PT1	1.000	1.000	1.000	sig.
CZ5 vs. PT4	0.959	0.887	0.999	sig.
CZ5 vs. RO1	0.756	0.725	0.847	sig.
CZ5 vs. SLO	0.210	0.076	0.379	sig.
CZ5 vs. SW1	-	-	-	-
DE2 vs. DE3	0.042	-0.011	0.156	n.s.
DE2 vs. EE1	0.042	-0.011	0.156	n.s.
DE2 vs. ES2	0.923	0.830	0.996	sig.
DE2 vs. ES4	0.715	0.578	0.868	sig.
DE2 vs. FR1	0.290	0.141	0.449	sig.
DE2 vs. FR6	0.034	-0.016	0.144	n.s.
DE2 vs. FR7	0.276	0.043	0.542	sig.
DE2 vs. CH4	0.923	0.830	0.996	sig.
DE2 vs. CH5	0.920	0.824	0.996	sig.
DE2 vs. IT1	0.681	0.556	0.809	sig.
DE2 vs. IT3	0.922	0.827	0.996	sig.
DE2 vs. PT1	0.922	0.827	0.996	sig.
DE2 vs. PT4	0.883	0.752	0.995	sig.
DE2 vs. RO1	0.683	0.579	0.796	sig.
DE2 vs. SLO	0.121	0.001	0.315	sig.
DE2 vs. SW1	0.009	-0.035	0.105	n.s.
DE3 vs. EE1	-	-	-	-
DE3 vs. ES2	1.000	1.000	1.000	sig.
DE3 vs. ES4	0.794	0.700	0.912	sig.
DE3 vs. FR1	0.359	0.224	0.511	sig.
DE3 vs. FR6	-	-	-	-
DE3 vs. FR7	0.410	0.190	0.654	sig.
DE3 vs. CH4	1.000	1.000	1.000	sig.
DE3 vs. CH5	1.000	1.000	1.000	sig.
DE3 vs. IT1	0.760	0.697	0.847	sig.
DE3 vs. IT3	1.000	1.000	1.000	sig.
DE3 vs. PT1	1.000	1.000	1.000	sig.
DE3 vs. PT4	0.960	0.889	0.999	sig.
DE3 vs. RO1	0.760	0.729	0.849	sig.
DE3 vs. SLO	0.214	0.079	0.384	sig.
DE3 vs. SW1	-	-	-	-
EE1 vs. ES2	1.000	1.000	1.000	sig.
EE1 vs. ES4	0.794	0.700	0.912	sig.
EE1 vs. FR1	0.359	0.224	0.511	sig.
EE1 vs. FR6	-	-	-	-
EE1 vs. FR7	0.410	0.190	0.654	sig.
EE1 vs. CH4	1.000	1.000	1.000	sig.
EE1 vs. CH5	1.000	1.000	1.000	sig.
EE1 vs. IT1	0.760	0.697	0.847	sig.
EE1 vs. IT3	1.000	1.000	1.000	sig.
EE1 vs. PT1	1.000	1.000	1.000	sig.

Pairs of locations	*FST*	Lower 95%CI	Upper 95%CI	Significance
EE1 vs. PT4	0.960	0.889	0.999	sig.
EE1 vs. RO1	0.760	0.729	0.849	sig.
EE1 vs. SLO	0.214	0.079	0.384	sig.
EE1 vs. SW1	-	-	-	-
ES2 vs. ES4	0.794	0.700	0.912	sig.
ES2 vs. FR1	0.654	0.617	0.692	sig.
ES2 vs. FR6	1.000	1.000	1.000	sig.
ES2 vs. FR7	0.750	0.701	0.822	sig.
ES2 vs. CH4	1.000	1.000	1.000	sig.
ES2 vs. CH5	1.000	1.000	1.000	sig.
ES2 vs. IT1	0.749	0.661	0.848	sig.
ES2 vs. IT3	1.000	1.000	1.000	sig.
ES2 vs. PT1	-	-	-	-
ES2 vs. PT4	0.000	-0.025	0.100	n.s.
ES2 vs. RO1	0.760	0.729	0.849	sig.
ES2 vs. SLO	0.780	0.705	0.882	sig.
ES2 vs. SW1	1.000	1.000	1.000	sig.
ES4 vs. FR1	0.492	0.404	0.590	sig.
ES4 vs. FR6	0.782	0.685	0.907	sig.
ES4 vs. FR7	0.489	0.356	0.646	sig.
ES4 vs. CH4	0.794	0.700	0.912	sig.
ES4 vs. CH5	0.786	0.690	0.909	sig.
ES4 vs. IT1	0.545	0.425	0.695	sig.
ES4 vs. IT3	0.790	0.695	0.910	sig.
ES4 vs. PT1	0.790	0.695	0.910	sig.
ES4 vs. PT4	0.753	0.622	0.879	sig.
ES4 vs. RO1	0.550	0.441	0.672	sig.
ES4 vs. SLO	0.570	0.438	0.719	sig.
ES4 vs. SW1	0.745	0.636	0.888	sig.
FR1 vs. FR6	0.347	0.214	0.499	sig.
FR1 vs. FR7	0.190	0.091	0.326	sig.
FR1 vs. CH4	0.640	0.579	0.690	sig.
FR1 vs. CH5	0.633	0.572	0.683	sig.
FR1 vs. IT1	0.465	0.397	0.550	sig.
FR1 vs. IT3	0.637	0.575	0.686	sig.
FR1 vs. PT1	0.650	0.613	0.689	sig.
FR1 vs. PT4	0.625	0.549	0.681	sig.
FR1 vs. RO1	0.469	0.416	0.544	sig.
FR1 vs. SLO	0.204	0.106	0.338	sig.
FR1 vs. SW1	0.311	0.182	0.462	sig.
FR6 vs. FR7	0.388	0.172	0.634	sig.
FR6 vs. CH4	1.000	1.000	1.000	sig.
FR6 vs. CH5	1.000	1.000	1.000	sig.
FR6 vs. IT1	0.747	0.682	0.838	sig.
FR6 vs. IT3	1.000	1.000	1.000	sig.
FR6 vs. PT1	1.000	1.000	1.000	sig.
FR6 vs. PT4	0.957	0.882	0.999	sig.
FR6 vs. RO1	0.748	0.716	0.841	sig.
FR6 vs. SLO	0.200	0.069	0.367	sig.
FR6 vs. SW1	-	-	-	-
FR7 vs. CH4	0.750	0.701	0.822	sig.
FR7 vs. CH5	0.739	0.689	0.814	sig.
FR7 vs. IT1	0.450	0.354	0.580	sig.
FR7 vs. IT3	0.745	0.695	0.818	sig.
FR7 vs. PT1	0.745	0.695	0.818	sig.
FR7 vs. PT4	0.698	0.572	0.808	sig.
FR7 vs. RO1	0.456	0.378	0.562	sig.
FR7 vs. SLO	0.142	0.011	0.356	sig.
FR7 vs. SW1	0.316	0.114	0.566	sig.
CH4 vs. CH5	-	-	-	-
CH4 vs. IT1	0.760	0.697	0.847	sig.
CH4 vs. IT3	-	-	-	-
CH4 vs. PT1	1.000	1.000	1.000	sig.
CH4 vs. PT4	0.960	0.889	0.999	sig.
CH4 vs. RO1	0.760	0.729	0.849	sig.
CH4 vs. SLO	0.780	0.705	0.882	sig.
CH4 vs. SW1	1.000	1.000	1.000	sig.
CH5 vs. IT1	0.751	0.687	0.841	sig.
CH5 vs. IT3	-	-	-	-
CH5 vs. PT1	1.000	1.000	1.000	sig.
CH5 vs. PT4	0.958	0.884	0.999	sig.
CH5 vs. RO1	0.752	0.720	0.844	sig.
CH5 vs. SLO	0.772	0.695	0.877	sig.
CH5 vs. SW1	1.000	1.000	1.000	sig.
IT1 vs. IT3	0.755	0.692	0.844	sig.
IT1 vs. PT1	0.745	0.656	0.845	sig.
IT1 vs. PT4	0.707	0.581	0.825	sig.
IT1 vs. RO1	0.516	0.432	0.636	sig.
IT1 vs. SLO	0.530	0.414	0.669	sig.
IT1 vs. SW1	0.705	0.633	0.808	sig.
IT3 vs. PT1	1.000	1.000	1.000	sig.
IT3 vs. PT4	0.959	0.887	0.999	sig.
IT3 vs. RO1	0.756	0.725	0.847	sig.
IT3 vs. SLO	0.776	0.700	0.879	sig.
IT3 vs. SW1	1.000	1.000	1.000	sig.
PT1 vs. PT4	-0.002	-0.026	0.097	n.s.
PT1 vs. RO1	0.756	0.725	0.847	sig.
PT1 vs. SLO	0.776	0.700	0.879	sig.
PT1 vs. SW1	1.000	1.000	1.000	sig.
PT4 vs. RO1	0.720	0.621	0.821	sig.
PT4 vs. SLO	0.740	0.630	0.874	sig.
PT4 vs. SW1	0.949	0.859	0.998	sig.
RO1 vs. SLO	0.540	0.446	0.672	sig.
RO1 vs. SW1	0.707	0.672	0.812	sig.
SLO vs. SW1	0.156	0.038	0.312	sig.
