# Supplementary material for: Three in One—Multiple Faunal Elements within an Endangered European Butterfly Species
Source: PLoS One. 2015 Nov 13;10(11):e0142282. doi: 10.1371/journal.pone.0142282 (PMC4643965; doi:10.1371/journal.pone.0142282)
Supplement: S2 Table — Groups defined in Table 2. (PDF) [file pone.0142282.s002.pdf]

Table S2.

Pairwise-group *Dest* (estimated) and *Fst* values for mtDNA sequences *E. aurinia*. Groups defined in Table 2.
(significance obtained by 10000 bootstraps)

D jost

Pairs of locations	*Dest*	Lower 95%CI	Upper 95%CI	Significance
mt1 vs. mt2	1.000	1.000	1.000	sig.
mt1 vs. mt3	1.000	1.000	1.000	sig.
mt1 vs. mt4	1.000	1.000	1.000	sig.
mt1 vs. mt5	1.000	1.000	1.000	sig.
mt1 vs. mt6	1.000	1.000	1.000	sig.
mt1 vs. mt7	0.945	0.823	1.002	sig.
mt1 vs. mt8	1.000	1.000	1.000	sig.
mt1 vs. mt9	1.000	1.000	1.000	sig.
mt2 vs. mt3	1.000	1.000	1.000	sig.
mt2 vs. mt4	1.000	1.000	1.000	sig.
mt2 vs. mt5	1.000	1.000	1.000	sig.
mt2 vs. mt6	1.000	1.000	1.000	sig.
mt2 vs. mt7	1.000	1.000	1.000	sig.
mt2 vs. mt8	1.000	1.000	1.000	sig.
mt2 vs. mt9	1.000	1.000	1.000	sig.
mt3 vs. mt4	0.283	0.107	0.597	sig.
mt3 vs. mt5	0.359	0.130	0.646	sig.
mt3 vs. mt6	0.287	0.084	0.593	sig.
mt3 vs. mt7	1.000	1.000	1.000	sig.
mt3 vs. mt8	0.203	0.003	0.558	sig.
mt3 vs. mt9	1.000	1.000	1.000	sig.
mt4 vs. mt5	0.325	0.173	0.494	sig.
mt4 vs. mt6	-0.005	-0.015	0.033	n.s.
mt4 vs. mt7	1.000	1.000	1.000	sig.
mt4 vs. mt8	0.188	0.108	0.319	sig.
mt4 vs. mt9	1.000	1.000	1.000	sig.
mt5 vs. mt6	0.328	0.165	0.502	sig.
mt5 vs. mt7	1.000	1.000	1.000	sig.
mt5 vs. mt8	0.284	0.117	0.481	sig.
mt5 vs. mt9	1.000	1.000	1.000	sig.
mt6 vs. mt7	1.000	1.000	1.000	sig.
mt6 vs. mt8	0.191	0.068	0.355	sig.
mt6 vs. mt9	1.000	1.000	1.000	sig.
mt7 vs. mt8	0.979	0.914	1.000	sig.
mt7 vs. mt9	1.000	1.000	1.000	sig.
mt8 vs. mt9	1.000	1.000	1.000	sig.

FST

Pairs of locations	*FST*	Lower 95%CI	Upper 95%CI	Significance
mt1 vs. mt2	0.8658	0.7885	0.9360	sig.
mt1 vs. mt3	0.8475	0.7824	0.9058	sig.
mt1 vs. mt4	0.6669	0.6385	0.6963	sig.
mt1 vs. mt5	0.7466	0.7028	0.7882	sig.
mt1 vs. mt6	0.7610	0.7205	0.8018	sig.
mt1 vs. mt7	0.8370	0.7545	0.9143	sig.
mt1 vs. mt8	0.8566	0.7830	0.9354	sig.
mt1 vs. mt9	0.8440	0.7869	0.8996	sig.
mt2 vs. mt3	0.4893	0.3621	0.6434	sig.
mt2 vs. mt4	0.5255	0.4617	0.5875	sig.
mt2 vs. mt5	0.4924	0.4044	0.5838	sig.
mt2 vs. mt6	0.5408	0.4637	0.6264	sig.
mt2 vs. mt7	0.5453	0.4207	0.6925	sig.
mt2 vs. mt8	0.5697	0.4386	0.7128	sig.
mt2 vs. mt9	0.5495	0.4420	0.6743	sig.
mt3 vs. mt4	0.1908	0.1046	0.3400	sig.
mt3 vs. mt5	0.1898	0.0866	0.3246	sig.
mt3 vs. mt6	0.1924	0.0913	0.3478	sig.
mt3 vs. mt7	0.4498	0.3474	0.5898	sig.
mt3 vs. mt8	0.1420	0.0108	0.3479	sig.
mt3 vs. mt9	0.4557	0.3786	0.5737	sig.
mt4 vs. mt5	0.2276	0.1458	0.3192	sig.
mt4 vs. mt6	-0.0103	-0.0209	0.0311	n.s.
mt4 vs. mt7	0.5060	0.4583	0.5682	sig.
mt4 vs. mt8	0.1629	0.1147	0.2273	sig.
mt4 vs. mt9	0.5087	0.4762	0.5588	sig.
mt5 vs. mt6	0.2267	0.1350	0.3194	sig.
mt5 vs. mt7	0.4646	0.3920	0.5579	sig.
mt5 vs. mt8	0.2041	0.1078	0.3250	sig.
mt5 vs. mt9	0.4687	0.4150	0.5425	sig.
mt6 vs. mt7	0.5151	0.4561	0.6015	sig.
mt6 vs. mt8	0.1711	0.0960	0.2682	sig.
mt6 vs. mt9	0.5186	0.4786	0.5906	sig.
mt7 vs. mt8	0.5303	0.4123	0.6704	sig.
mt7 vs. mt9	0.5155	0.4343	0.6382	sig.
mt8 vs. mt9	0.5400	0.4464	0.6631	sig.
