# Supplementary material for: Three in One—Multiple Faunal Elements within an Endangered European Butterfly Species
Source: PLoS One. 2015 Nov 13;10(11):e0142282. doi: 10.1371/journal.pone.0142282 (PMC4643965; doi:10.1371/journal.pone.0142282)
Supplement: S3 Table — (PDF) [file pone.0142282.s003.pdf]

Table S3.

Electrophoresis conditions for the different enzymes analysed for *Euphydryas aurinia*.

Enzyme	EC no.	Number of loci	Buffer	Homogenate applications	Running time (min)
6Pgdh	1.1.1.44	1	TM	2	50
Idh	1.1.1.42	2	TM	2	50
Pep (Phe-Pro)	3.4.11/13	1	TG	3	40
Fum	4.2.1.2	1	TG	3	40
Aat	2.6.1.1	2	TG	3	30
Hbdh	1.1.1.30	1	TG	2	30
G6pdh	1.1.1.49	1	TC	2	45
Me	1.1.1.40	1	TC	3	40
Mdh	1.1.1.37	2	TC	3	40
Mpi	5.3.1.8	1	TC	3	30
Gapdh	1.2.1.12	1	TC	3	45
Gpdh	1.1.1.8	1	TM	3	40
Pgi	5.3.1.9	1	TG	2	40
Pgm	5.4.2.2	1	TG	2	40
Acon	4.2.1.3	1	TG	4	35

TC: Tris-citrate, pH = 8.2 [39]; TG: Tris-glycine, pH = 8.5 [40]; TM: Tris-maleic acid, pH = 7.0 (adapted from [39]). Electrophoreses were run at 200 V.
